# Dietary quality across the life course and metabolic syndrome: evidence from two decades of follow-up in rural China young adults

**DOI:** 10.3389/fpubh.2026.1826210

**Published:** 2026-06-23

**Authors:** Liang Wang, Yingze Zhu, Jiayu Shan, Jialu Li, Zhonghai Zhu, Wenfang Yang, Suying Chang, Lingxia Zeng, Xiaojing Bai

**Affiliations:** 1 Department of Epidemiology and Biostatistics, School of Public Health, Xi’an Jiaotong University Health Science Center, Xi'an, Shaanxi, China; 2 Center for Chronic Disease Control and Prevention, Global Health Institution, Xi’an Jiaotong University, Xi'an, China; 3 Key Laboratory for Disease Prevention and Control and Health Promotion of Shaanxi Province, Xi'an, China; 4 Department of Obstetrics and Gynecology, Maternal and Child Health Center, The First Affiliated Hospital of Xi'an Jiaotong University, Xi'an, China; 5 Child Health Development Section, United Nations Children's Fund, Office for China, Beijing, China; 6 Nutrition and Food Safety Engineering Research Center of Shaanxi Province, Xi'an, Shaanxi, China; 7 Department of Urology, The First Affiliated Hospital of Xi'an Jiaotong University, Xi'an, Shaanxi, China

**Keywords:** birth cohort study, EAT-Lancet score, metabolic syndrome, trajectories, young adults

## Abstract

**Background:**

The global prevalence of metabolic syndrome (MetS) among young adults has reached 17.4%. We examined associations of three dietary indices and life-course dietary quality trajectories with MetS and its components using a birth cohort.

**Methods:**

Participants from rural western China were followed at four time points: infancy, school age, adolescence, and young adulthood (mean age 20 years). Three dietary indices—Dietary Diversity Score (DDS), healthy Plant-based Diet Index (h-PDI), and EAT-Lancet score—were compared. Group-based trajectory modeling (GBTM) characterized dietary quality trajectories across the four stages.

**Results:**

Among 741 young adults (53% male), 29 (5.1%) had MetS, 217 (38.2%) had at least one metabolic abnormality, and 122 (21.1%) were overweight or obese. Higher DDS was inversely associated with overweight/obesity (T3 vs. T1: OR 0.26, 95% CI 0.10–0.69) and high waist-to-height ratio (WHtR) (OR 0.26, 95% CI 0.09–0.73). Higher h-PDI was positively associated with high WHtR (OR 3.01, 95% CI 1.11–8.15). The EAT-Lancet score was inversely associated with overweight/obesity (OR 0.34, 95% CI 0.15–0.78). After stratified Benjamini-Hochberg correction, these four associations remained significant (adjusted *P* [*Q*] values 0.016–0.046). Among 298 participants with complete dietary data, two trajectories were identified: increasing (53.0%) and declining (47.0%). The increasing trajectory was associated with lower odds of overweight/obesity (OR 0.34, 95% CI 0.15–0.77).

**Conclusion:**

In this rural Chinese cohort, higher DDS and EAT-Lancet were associated with lower odds of overweight/obesity after FDR correction. An increasing dietary trajectory from infancy to young adulthood was also protective. All three indices showed similar discriminative ability. These findings support life-course dietary health promotion.

## 1 Introduction

Metabolic syndrome (MetS) is a cluster of interrelated metabolic abnormalities, including elevated cholesterol, high blood pressure, impaired glucose regulation, and—as is now recognized—central obesity as a key component (1, 2). The global prevalence of metabolic problems continues to rise (3). Among young adults, the prevalence of MetS has reached 17.4%, with increasing incidence documented in both developed and developing countries (4). Early-onset MetS may lead to serious long-term health consequences.

Dietary modification is a key strategy for the prevention and management of MetS (5). Healthy dietary practices may contribute to the stabilization or improvement of numerous disease-related biomarkers (6). Accurate dietary assessment is essential to the provision of effective dietary guidance (7, 8). Although multiple methods exist to evaluate dietary patterns, developing a brief and practical tool tailored to young adults remains an important research priority (9).

Dietary indices are widely used because of their efficiency and ease of application (8). The Food Frequency Questionnaire (FFQ) is commonly employed in population-based research. The Dietary Diversity Score (DDS), the healthful Plant-based Diet Index (h-PDI), and EAT-Lancet score are all constructed from FFQ-derived data. The h-PDI has demonstrated health-promoting effects in developed countries; however, its strict limitations on animal protein intake remain a subject of debate (10). The EAT-Lancet score offers a more balanced approach to the regulation of animal protein consumption. Several studies have reported associations between these indices and MetS (11–13), but their validity must be examined across diverse populations and life stages (14–16). Most longitudinal studies of diet quality and MetS have focused on a single developmental stage or have examined only specific nutrients: Chia et al. (17) used principal component analysis to derive composite lifestyle patterns across ages 2–8; Kerr et al. (18) assessed diet quality trajectories using the Dietary Guideline Index for Children and Adolescents from ages 2 to 12; Hassannejad et al. (19) identified dietary patterns via exploratory factor analysis at baseline with 13-year follow-up; and Dam et al. (20) examined only vitamin K intake at baseline. Collectively, these studies examined dietary exposures at limited developmental stages or with single indices, leaving the comparative performance of multiple *a priori* dietary indices and their trajectories across the full life course unexplored.

The present study used up to 20 years of follow-up data from a birth cohort in rural western China. We focused on overweight/obesity and elevated blood pressure (the earliest onset metabolic disorders) as primary outcomes; MetS and other individual components served as secondary outcomes. An exploratory aim was to examine how dietary patterns from infancy through early adulthood relate to metabolic dysfunction in early adulthood. Our findings may inform the design of dietary interventions during critical developmental periods.

## 2 Methods

### 2.1 Study design and participants

This birth cohort study originated from a double-blind, cluster-randomized trial conducted between 2002 and 2006 in two rural counties of western China (ISRCTN08850194). The original trial has been described in detail elsewhere (21). Children born during the trial were followed at four time points: infancy (2004–2008), school age (2012–2013), early adolescence (2016), and young adulthood (2024).

**Figure 1:**
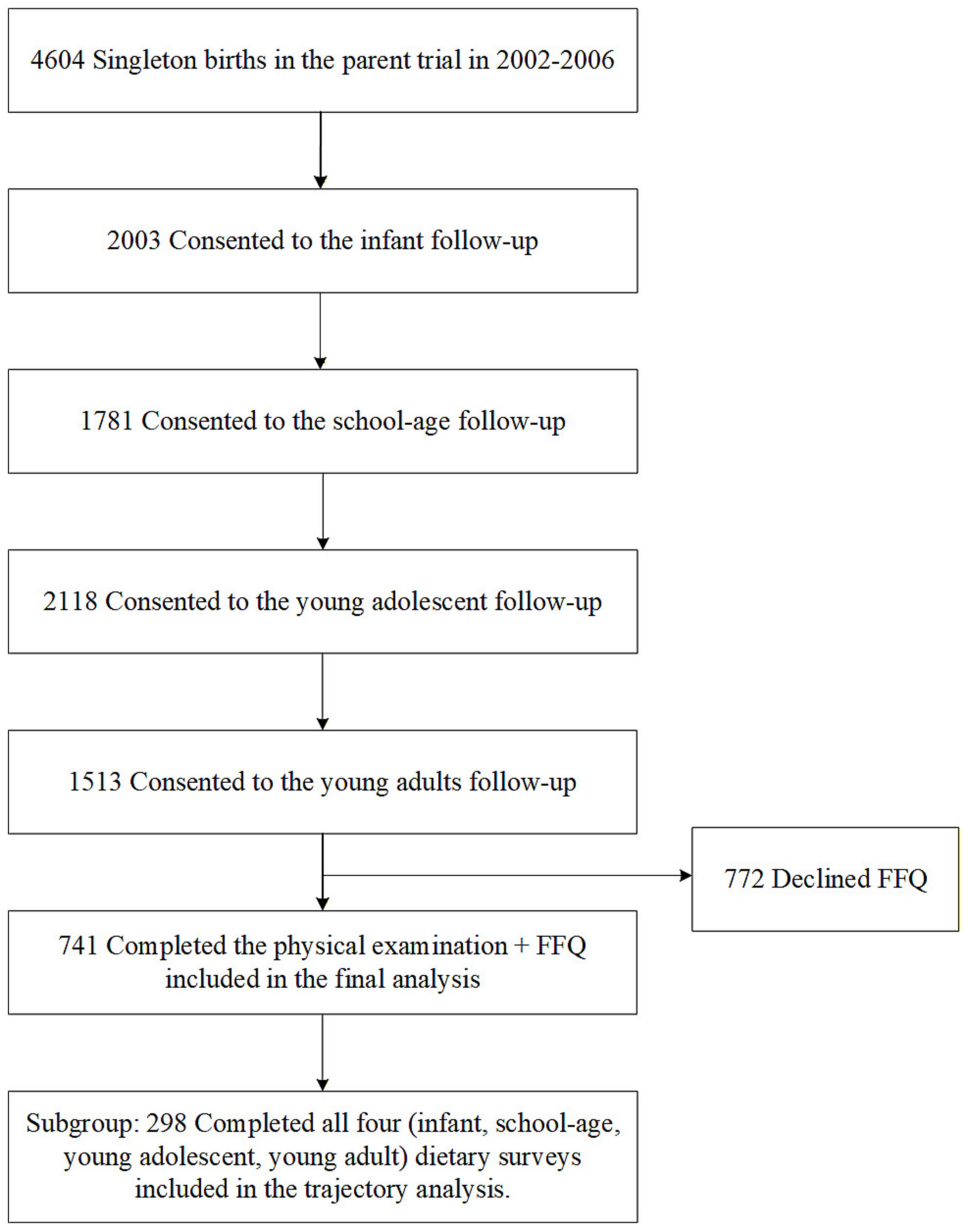
Flowchart of participant selection and follow-up. The original trial was conducted between 2002 and 2006 in two rural counties of western China (ISRCTN08850194). Of 1,513 singleton live births from the trial, follow-up was conducted at four time points: infancy (2004–2008), school age (2012–2013), early adolescence (2016), and young adulthood (2024). A total of 741 participants completed the physical examination and food frequency questionnaire (FFQ) at the young adult wave and were included in the cross-sectional dietary index analyses. Of these, 298 participants with complete dietary data across all four life stages were included in the trajectory analysis.

Exclusion criteria included multiple pregnancies, congenital malformations, severe genetic disorders, relocation out of the study area, and death. The original trial comprised 4,604 singleton live births. At each follow-up wave, all surviving members of the original cohort were re-contacted, regardless of whether they had participated in prior waves. The numbers of participants who completed each follow-up wave were: infancy (2004–2008, *n* = 2,003); school age (2012–2013, *n* = 1,781); early adolescence (2016, *n* = 2,118); and young adulthood (2024, *n* = 1,513). Of the 1,513 who agreed to the young adult wave, declined to complete the FFQ, and 741 completed both the physical examination and the FFQ, constituting the cross-sectional dietary index analysis sample. Of these 741 respondents, 443 had incomplete dietary data across the four stages, leaving 298 participants (40.2%) with complete dietary data for the trajectory analysis (Figure 1). Ethical approval for the original trial and earlier follow-up waves has been reported previously (Nos. 2,002,001, 2,016,345, and 2,024,223). For the current follow-up, approval was obtained from the Ethics Committee of Xi’an Jiaotong University Health Science Center, and written informed consent was collected from all young adult participants.

### 2.2 Sample size and power

The sample size for the cross-sectional dietary index analyses was estimated based on the prevalence of central obesity (44% in the lowest DDS quartile) and a projected odds ratio of 2.20 (*α* = 0.05, power = 0.80) (22). PASS software indicated that 494 participants were required, allowing for 10% attrition. The final analytic sample comprised 741 participants, satisfying this requirement.

For the trajectory analysis (*N* = 298), a formal *a priori* power calculation was not feasible because GBTM lacks a closed-form power formula. We therefore evaluated the adequacy of this subsample through two complementary approaches. First, we conducted a post-hoc power analysis for the comparison between trajectory groups and metabolic outcomes. Using the observed prevalence in the declining group and the adjusted OR from the logistic regression models, we derived the implied prevalence in the increasing group and computed power via two-proportion z-tests (two-sided *α* = 0.05; Supplementary Table S11). The power was 0.88 for overweight/obesity (OR = 0.34) but only 0.32 for MetS (OR = 0.35), indicating adequate power for common outcomes but insufficient power for rare outcomes. Second, methodological research has shown that GBTM can generate spurious findings when trajectories are continuously distributed, and that BIC alone is insufficient for model selection (23); trajectory groups should be interpreted as statistical approximations rather than evidence of distinct subpopulations (24). Our 2-group model was selected as the most parsimonious solution, with both groups being substantial (*n* = 131 and *n* = 102, each >40% of the sample).

To address potential selection bias from the reduction to 298 participants, an attrition analysis and inverse probability weighting were applied (see Section 2.5 for details; Supplementary Tables S4, S5).

### 2.3 Outcomes

#### 2.3.1 Metabolic syndrome

MetS components were defined according to *the Guideline for the Prevention and Treatment of Diabetes Mellitus in China (2024 edition)* issued by the Chinese Diabetes Society (25). The diagnostic criteria were as follows:

i) Abdominal obesity: waist circumference ≥90 cm for men and ≥85 cm for women.
ii) Hyperglycemia: fasting blood glucose ≥6.1 mmol/L (this study used glycated hemoglobin (HbA1c) ≥ 6.5% as a more stable indicator).
iii) Hypertension: blood pressure (BP) ≥ 130/85 mmHg or previously diagnosed and treated hypertension.
iv) Dyslipidemia: fasting triglyceride ≥1.70 mmol/L.
v) Low high-density lipoprotein cholesterol (HDL-C): fasting HDL-C < 1.04 mmol/L.

MetS was defined as the presence of three or more of these components. The presence of any single abnormality was defined as having one metabolic problem.

Waist circumference (WC) was measured twice at the midpoint of the abdomen with the participant in a standing position. Weight was measured to the nearest 0.1 kg using a bioelectrical impedance device (BC420, Tanita Corporation). Height was measured to the nearest millimeter using a stadiometer (SZG-210, Shanghai JWFU). Shoes and heavy clothing were removed prior to measurement. Body mass index (BMI) was calculated as weight divided by height squared (kg/m^2^). Overweight and obesity were classified according to the Chinese national adult criteria (26). Waist-to-height ratio (WHtR) was computed as WC divided by standing height (27).

Blood samples were collected after at least 8 h of fasting. Plasma triglycerides and HDL-C were analyzed using enzymatic methods. Abnormal blood glucose was defined using HbA1c, with HbA1c ≥ 6.5% considered elevated.

#### 2.3.2 Hypertension/elevated blood pressure

Blood pressure was measured twice by trained staff using validated electronic devices under resting conditions, with a five-minute interval between measurements. The mean of the two readings was recorded. Blood pressure assessment followed the Chinese Guidelines for the Prevention and Treatment of Hypertension (2024 revision) (28).

### 2.4 Exposure

#### 2.4.1 Dietary assessment

Dietary intake during the previous 6 months was assessed using a simplified FFQ. FFQ has been previously applied and validated in rural Chinese populations (29, 30). The questionnaire covered nine food groups commonly consumed in China: meat, fish, eggs, milk, grains, beans, vegetables, fruits, and high-fat items. The DDS ranged from 1 to 9, with higher scores indicating greater dietary diversity (31). Because staple grains are consumed daily by nearly all individuals in China, 1 point was assigned for grains. For the remaining food groups, intake was scored 1 point if the item was consumed at least “2–4 times per week,” and 0 if consumed less frequently.

The h-PDI assigned positive scores to healthful plant-based foods and negative scores to less healthful plant-based foods and animal-derived foods (32). Seventeen common food groups were included:

Healthful plant foods: nuts, tea, fruits, vegetables, soy products, whole grains.

Less healthful plant foods: potatoes, fruit juice, refined grains, sweets and desserts.

Animal-based foods: miscellaneous animal-based foods, sugar-sweetened beverages, dairy, eggs, fish and aquatic products, meat, animal fats.

Scoring was based on frequency of consumption: “≥1 time/day” = 5 points; “5–6 times/week” = 4 points; “2–4 times/week” = 3 points; “1 time/week” or “1–3 times/month” = 2 points; “<1 time/month or rarely” = 1 point.

We also constructed an EAT-Lancet score based on 14 key recommendations. One point was assigned for each recommendation met, yielding a total score ranging from 0 to 14 (33).

In earlier phases of this study, we created the infant and young child feeding (IYCF) index by summing related variables in accordance with WHO recommendations (34). For example, exclusive breastfeeding for 6 months was scored as 4, while shorter durations received lower scores (1 for <1 month, 2 for 2–3 months, 3 for 4–5 months). Infants breastfed for longer than 7 months were assigned a score of 1, under the assumption that delayed introduction of complementary feeding reduces benefits. Scores were grouped into tertiles to indicate the quality of feeding practices.

#### 2.4.2 Covariates

Covariates were selected *a priori* using a directed acyclic graph (DAG) informed by the life-course nutritional epidemiology literature (Supplementary Figure S1). The original trial treatment group (folic acid, iron-folic acid, or multiple micronutrients) was included in all models, given the significant association between prenatal supplementation and both birth weight and gestational duration. The minimally sufficient adjustment set comprised gender, treatment group, parental education and occupation, household wealth index, birth weight, gestational age, infant feeding pattern, age at follow-up, moderate physical activity, sleep quality, smoking history, and employment status. Socioeconomic position was captured by parental education, occupation, and household wealth. Early-life nutritional conditions were represented by birth weight, gestational age, and feeding pattern. For the dietary index analyses (Table 1), total energy intake was adjusted for, given its correlation with dietary index scores. The trajectory analyses (Table 2) did not include energy intake, as trajectory groups reflect dietary quality patterns rather than absolute intake. Parental occupation was classified as farmer versus other. Parental education was categorized as less than 3 years, primary school, secondary school, or high school and above. Gender, age, smoking history, school enrollment, moderate physical activity, and sleep quality (very good, good, poor, or very poor) were collected through structured questionnaires. Total energy intake was derived from the FFQ and included as a continuous covariate.

**Table 1:** Association between three dietary indices and metabolic outcomes.

Dietary index	Outcome	Method	OR (95% CI)	*p*	*p* trend
DDS	MetS	Low		Ref.	0.135
		Medium	0.363 (0.091–1.447)	0.151	
		High	0.340 (0.080–1.448)	0.145	
	Metabolic problems	Low		Ref.	0.525
		Medium	0.481 (0.203–1.142)	0.097	
		High	0.696 (0.299–1.624)	0.402	
	Overweight or obesity	Low		Ref.	0.007
		Medium	0.438 (0.173–1.106)	0.081	
		High	0.262 (0.099–0.691)	0.007	
	Elevated BP/hypertension	Low		Ref.	0.814
		Medium	0.881 (0.362–2.142)	0.779	
		High	0.891 (0.370–2.146)	0.796	
	WHtR	Low		Ref.	0.010
		Medium	0.291 (0.105–0.808)	0.018	
		High	0.259 (0.092–0.728)	0.010	
h-PDI	MetS	Low		Ref.	0.697
		Medium	0.550 (0.167–1.819)	0.328	
		High	1.034 (0.267–4.004)	0.961	
	Metabolic problems	Low		Ref.	0.908
		Medium	0.679 (0.360–1.280)	0.231	
		High	1.279 (0.590–2.773)	0.533	
	Overweight or obesity	Low		Ref.	0.677
		Medium	0.919 (0.441–1.914)	0.821	
		High	1.334 (0.533–3.335)	0.538	
	Elevated BP/hypertension	Low		Ref.	0.513
		Medium	1.106 (0.580–2.112)	0.759	
		High	1.321 (0.581–3.001)	0.506	
	WHtR	Low		Ref.	0.030
		Medium	1.683 (0.717–3.953)	0.232	
		High	3.005 (1.108–8.152)	0.031	
EAT-Lancet score	MetS	Low		Ref.	0.477
		Medium	0.709 (0.202–2.485)	0.591	
		High	0.640 (0.166–2.477)	0.519	
	Metabolic problems	Low		Ref.	0.375
		Medium	0.701 (0.350–1.404)	0.316	
		High	0.733 (0.371–1.449)	0.372	
	Overweight or obesity	Low		Ref.	0.009
		Medium	0.339 (0.145–0.795)	0.013	
		High	0.340 (0.148–0.778)	0.011	
	Elevated BP/hypertension	Low		Ref.	0.875
		Medium	0.921 (0.448–1.893)	0.823	
		High	0.944 (0.466–1.911)	0.873	
	WHtR	Low		Ref.	0.983
		Medium	1.162 (0.466–2.898)	0.748	
		High	0.990 (0.397–2.470)	0.983	

OR, odds ratio; CI, confidence interval; MetS, metabolic syndrome; BP, blood pressure; WHtR, waist-to-height ratio; DDS, Dietary Diversity Score; h-PDI, healthful Plant-based Diet Index. Reference category: Tertile 1 (lowest dietary quality). MetS was analyzed using Firth penalized logistic regression due to rare events; all other outcomes were analyzed using standard logistic regression. *P* for trend was calculated by modeling the median value of each dietary index within tertiles as a continuous variable. All models were adjusted for parental occupation, parental education, household wealth, treatment group, birth weight, moderate physical activity, sleep quality, gender, smoking history, employment status, age, and energy intake.

**Table 2:** Association between diet quality trajectory and metabolic outcomes.

Outcome	Method	Increasing vs. declining OR (95% CI)	*p*-value
MetS	Declining		Ref.
	Increasing	0.35 (0.07–1.70)	0.195
Metabolic problems	Declining		Ref.
	Increasing	1.24 (0.67–2.30)	0.490
Overweight or obesity	Declining		Ref.
	Increasing	0.34 (0.15–0.77)	0.009
Elevated BP/hypertension	Declining		Ref.
	Increasing	0.99 (0.53–1.85)	0.972
WHtR	Declining		Ref.
	Increasing	0.44 (0.18–1.06)	0.067

OR, odds ratio; CI, confidence interval; MetS, metabolic syndrome; BP, blood pressure; WHtR, waist-to-height ratio. Reference group: Declining trajectory. The Increasing trajectory group was compared against the Declining trajectory group. MetS was analyzed using Firth penalized logistic regression due to rare events; all other outcomes were analyzed using standard logistic regression. All models were adjusted for parental occupation, parental education, household wealth, treatment group, birth weight, moderate physical activity, sleep quality, gender, smoking history, employment status, and age.

### 2.5 Statistical analysis

Binary variables were expressed as *n* (%), and continuous variables as mean ± standard deviation (SD). Categorical variables were compared using chi-square tests. Continuous variables were compared using the Mann–Whitney U test for two-group comparisons and the Kruskal–Wallis test for comparisons among three or more groups. Standardized mean differences (SMD) were calculated to compare baseline characteristics between groups, with SMD > 0.1 considered indicative of a meaningful imbalance.

First, to examine the association between current diet and metabolic outcomes, DDS was categorized as T1 (1–4), T2 (5–6), and T3 (7–9), and the h-PDI and EAT-Lancet scores were divided into tertiles, with T1 (lowest) serving as the reference category.

To capture longitudinal changes in dietary quality, we applied group-based trajectory modeling (GBTM) across four developmental stages: infancy, school age, adolescence, and young adulthood. At each stage, the dietary index most appropriate for that period was used: the IYCF index in infancy and DDS at the three later stages. These two indices assess related but distinct constructs—IYCF captures infant feeding adequacy, whereas DDS reflects dietary variety. To enable comparability across stages, all dietary measures were converted to the standardized dietary quality scores (*z*-score) within the trajectory sample before model fitting, so that each time point contributed a standardized measure of relative dietary quality regardless of the original scoring scale. This approach is consistent with prior GBTM studies that integrate developmentally appropriate instruments through within-sample standardization. We acknowledge that the shift from IYCF to DDS remains a potential source of measurement heterogeneity. To test whether the dietary effects operate continuously across the life course or act through a sensitive period, we entered dietary quality at all four stages—infancy (IYCF), school age (DDS), adolescence (DDS), and young adulthood (DDS, h-PDI, or EAT-Lancet score)—into the same fully adjusted OLS model as independent exposures. Group trajectory models were fitted for 1–4 classes, and the optimal number of classes was chosen via the Bayesian Information Criterion (BIC) (Supplementary Table S6). To address potential selection bias from loss to follow-up (298 of 741 participants had complete dietary data across all four stages), inverse probability weighting (IPW) was applied (Supplementary Table S5).

Logistic regression models were applied to estimate odds ratio (OR) and 95% confidence interval (CI) for the associations between dietary measures and metabolic outcomes. Because the number of MetS cases was small (*n* = 29), Firth penalized logistic regression was used for analyses with MetS as the outcome. For all other binary outcomes, standard logistic regression was employed. Potential effect modification by gender was assessed by including a product term (dietary index × gender or trajectory group × gender) in the fully adjusted logistic regression models. The statistical significance of each interaction term was evaluated using the Wald test. No significant interactions were observed (all *p* > 0.05; Supplementary Table S7), and therefore all analyses are presented for the pooled sample.

IPW was applied to address selection bias. Stabilized weights were derived from logistic regression predicting trajectory subsample inclusion using gender, treatment group, parental education and occupation, birth weight, and age. Variables with >40% missingness were excluded from the weighting model but retained in outcome models. Weights were truncated at the 1st and 99th percentiles. After weighting, all standardized mean differences fell below 0.02 (Supplementary Table S5). IPW relies on the missing-at-random assumption; given the substantial attrition, unmeasured factors may drive dropout, and the data may be missing not at random. Baseline comparisons between included and excluded participants are presented in Supplementary Table S4.

The Benjamini–Hochberg false discovery rate (FDR) procedure was applied to the cross-sectional dietary index analyses (3 indices × 5 outcomes = 15 tests). FDR-adjusted *p*-values(*Q*) are reported in Supplementary Table S8. The trajectory analysis (5 comparisons) was treated as a separate family of tests, as it addresses a distinct research question. As trajectory analysis was conducted as an exploratory analysis on a subsample, we did not apply corrections for multiple comparisons.

Statistical significance was set at a two-sided *p* < 0.05. All analyses were performed using Stata version 15 (StataCorp LLC).

## 3 Results

### 3.1 Sociodemographic characteristics

Table 3 presents participant characteristics stratified by tertiles of DDS, h-PDI, and EAT-Lancet scores. The final analytic sample included 741 young adults, of whom 393 (53.0%) were male and 348 (47.0%) were female. The mean age was 19.5 years (SD = 0.8). Most mothers (53.7%) and fathers (65.4%) had completed junior high school. The majority worked as farmers (mothers: 86.6%; fathers: 78.2%).

**Table 3:** Participant characteristics by dietary index category (*N* = 741).

Characteristic	Total	DDS					h-PDI					EAT-Lancet score				
		T1 (Low)	T2 (Medium)	T3 (High)	*p* ^1^	ASD	T1 (Low)	T2 (Medium)	T3 (High)	*p* ^1^	ASD	T1 (Low)	T2 (Medium)	T3 (High)	*p* ^1^	ASD
Maternal age	44.5 (4.4)	44.8 (4.4)	44.9 (4.5)	43.8 (4.1)	0.032	0.250	44.5 (4.2)	44.8 (4.5)	44.0 (4.4)	0.240	0.127	44.7 (4.6)	44.9 (4.3)	43.7 (4.1)	0.031	0.242
Maternal educational attainment					<0.001	0.138				0.026	0.075				0.326	0.059
<3 y	39 (5.3)	14 (5.5)	7 (3.1)	18 (6.9)			10 (3.8)	11 (4.1)	18 (8.7)			12 (5.2)	14 (5.1)	13 (5.6)		
Primary	204 (27.6)	78 (30.6)	64 (28.6)	62 (23.9)			64 (24.4)	85 (31.6)	55 (26.6)			74 (32.2)	68 (24.7)	62 (26.6)		
Secondary	396 (53.7)	144 (56.5)	128 (57.1)	124 (47.9)			143 (54.6)	139 (51.7)	114 (55.1)			112 (48.7)	162 (58.9)	122 (52.4)		
High school or higher	99 (13.4)	19 (7.5)	25 (11.2)	55 (21.2)			45 (17.2)	34 (12.6)	20 (9.7)			32 (13.9)	31 (11.3)	36 (15.5)		
Paternal educational attainment					<0.001	0.172				0.007	0.112				0.314	0.049
<3 y	5 (0.7)	1 (0.4)	3 (1.3)	1 (0.4)			2 (0.8)	2 (0.7)	1 (0.5)			0 (0.0)	3 (1.1)	2 (0.9)		
Primary	78 (10.5)	34 (13.3)	22 (9.8)	22 (8.5)			14 (5.3)	34 (12.6)	30 (14.4)			29 (12.6)	25 (9.1)	24 (10.2)		
Secondary	484 (65.4)	179 (69.9)	156 (69.3)	149 (57.5)			172 (65.4)	171 (63.6)	141 (67.8)			150 (65.2)	189 (68.7)	145 (61.7)		
High school or higher	173 (23.4)	42 (16.4)	44 (19.6)	87 (33.6)			75 (28.5)	62 (23.0)	36 (17.3)			51 (22.2)	58 (21.1)	64 (27.2)		
Maternal occupation					0.009	0.092				0.468	0.038				0.932	0.009
Farmer	639 (86.6)	232 (91.0)	196 (87.1)	211 (81.8)			223 (85.1)	231 (86.2)	185 (88.9)			200 (87.0)	238 (86.9)	201 (85.9)		
Other	99 (13.4)	23 (9.0)	29 (12.9)	47 (18.2)			39 (14.9)	37 (13.8)	23 (11.1)			30 (13.0)	36 (13.1)	33 (14.1)		
Paternal occupation					<0.001	0.149				0.173	0.055				0.375	0.050
Farmer	579 (78.2)	217 (84.8)	181 (80.4)	181 (69.9)			203 (77.2)	204 (75.8)	172 (82.7)			187 (81.3)	213 (77.5)	179 (76.2)		
Other	161 (21.8)	39 (15.2)	44 (19.6)	78 (30.1)			60 (22.8)	65 (24.2)	36 (17.3)			43 (18.7)	62 (22.5)	56 (23.8)		
Household wealth level					0.006	0.106				0.051	0.094				0.770	0.040
Low	154 (32.2)	69 (40.8)	44 (31.0)	41 (24.4)			43 (25.7)	60 (34.1)	51 (37.5)			57 (35.2)	51 (28.7)	46 (33.1)		
Medium	161 (33.6)	58 (34.3)	46 (32.4)	57 (33.9)			55 (32.9)	56 (31.8)	50 (36.8)			51 (31.5)	63 (35.4)	47 (33.8)		
High	164 (34.2)	42 (24.9)	52 (36.6)	70 (41.7)			69 (41.3)	60 (34.1)	35 (25.7)			54 (33.3)	64 (36.0)	46 (33.1)		
Paternal age	47.9 (3.8)	48.2 (3.8)	48.1 (4.0)	47.3 (3.6)	0.053	0.257	47.8 (3.8)	48.1 (3.8)	47.6 (4.0)	0.636	0.054	48.2 (4.0)	47.9 (3.9)	47.4 (3.6)	0.162	0.222
Gender					0.373	0.060				0.447	0.009				0.001	0.164
Male	393 (53.0)	127 (49.6)	122 (54.0)	144 (55.6)			143 (54.4)	135 (50.0)	115 (55.3)			144 (62.6)	140 (50.9)	109 (46.2)		
Female	348 (47.0)	129 (50.4)	104 (46.0)	115 (44.4)			120 (45.6)	135 (50.0)	93 (44.7)			86 (37.4)	135 (49.1)	127 (53.8)		
Prenatal supplementation group					0.383	0.027				0.523	0.003				0.373	0.021
Folic	240 (32.4)	75 (29.3)	78 (34.5)	87 (33.6)			90 (34.2)	79 (29.3)	71 (34.1)			75 (32.6)	84 (30.5)	81 (34.3)		
Folic + Iron	254 (34.3)	87 (34.0)	83 (36.7)	84 (32.4)			84 (31.9)	103 (38.1)	67 (32.2)			82 (35.7)	87 (31.6)	85 (36.0)		
Multiple micronutrients	247 (33.3)	94 (36.7)	65 (28.8)	88 (34.0)			89 (33.8)	88 (32.6)	70 (33.7)			73 (31.7)	104 (37.8)	70 (29.7)		
Young adults age	19.5 (0.8)	19.4 (0.8)	19.5 (0.8)	19.5 (0.9)	0.666	0.022	19.6 (0.8)	19.4 (0.8)	19.4 (0.9)	0.007	0.223	19.5 (0.9)	19.5 (0.8)	19.5 (0.8)	0.842	0.053
Birth weight, g	3201.2 (410.5)	3223.0 (409.3)	3246.4 (429.1)	3140.0 (388.8)	0.012	0.208	3166.5 (375.1)	3242.0 (429.9)	3192.2 (425.0)	0.106	0.064	3237.7 (387.9)	3193.7 (419.8)	3174.3 (420.1)	0.246	0.157
In school					0.138	0.056				0.009	0.072				0.026	0.085
No	637 (86.2)	211 (82.7)	198 (87.6)	228 (88.4)			230 (87.5)	242 (89.6)	165 (80.1)			188 (82.1)	235 (85.8)	214 (90.7)		
Yes	102 (13.8)	44 (17.3)	28 (12.4)	30 (11.6)			33 (12.5)	28 (10.4)	41 (19.9)			41 (17.9)	39 (14.2)	22 (9.3)		
Sleep quality level					0.324	0.022				0.320	0.085				0.587	0.025
1	185 (25.0)	61 (23.8)	50 (22.1)	74 (28.6)			62 (23.6)	65 (24.1)	58 (27.9)			58 (25.2)	60 (21.8)	67 (28.4)		
2	428 (57.8)	144 (56.2)	144 (63.7)	140 (54.1)			159 (60.5)	161 (59.6)	108 (51.9)			128 (55.7)	167 (60.7)	133 (56.4)		
3	112 (15.1)	44 (17.2)	29 (12.8)	39 (15.1)			34 (12.9)	39 (14.4)	39 (18.8)			37 (16.1)	43 (15.6)	32 (13.6)		
4	16 (2.2)	7 (2.7)	3 (1.3)	6 (2.3)			8 (3.0)	5 (1.9)	3 (1.4)			7 (3.0)	5 (1.8)	4 (1.7)		
Exercise in the past 7 days					<0.001	0.205				0.161	0.087				0.525	0.041
Yes	290 (39.5)	76 (29.8)	86 (38.4)	128 (50.0)			112 (42.9)	107 (40.1)	71 (34.3)			83 (36.4)	111 (40.8)	96 (40.9)		
No	445 (60.5)	179 (70.2)	138 (61.6)	128 (50.0)			149 (57.1)	160 (59.9)	136 (65.7)			145 (63.6)	161 (59.2)	139 (59.1)		
Young adults weight, kg	61.7 (12.6)	62.7 (13.6)	60.8 (11.7)	61.4 (12.4)	0.335	0.103	61.2 (12.7)	61.1 (12.0)	63.2 (13.4)	0.244	0.152	65.5 (14.3)	60.4 (11.9)	59.8 (11.2)	<0.001	0.441
Young adults height, cm	168.9 (8.3)	168.8 (8.4)	168.1 (8.2)	169.7 (8.4)	0.178	0.108	169.2 (8.2)	168.6 (8.2)	168.8 (8.9)	0.771	0.049	170.8 (7.8)	167.9 (8.5)	168.3 (8.3)	0.001	0.316
Waist circumference, cm	76.0 (10.8)	77.4 (11.9)	74.6 (9.6)	75.9 (10.5)	0.052	0.130	75.1 (10.3)	75.8 (10.9)	77.7 (11.2)	0.077	0.240	78.6 (11.6)	75.5 (11.2)	74.3 (9.2)	<0.001	0.413
Biceps circumference, cm	94.5 (8.1)	95.2 (8.6)	93.7 (7.5)	94.5 (8.2)	0.249	0.076	93.8 (7.9)	94.3 (8.0)	95.9 (8.5)	0.051	0.254	96.2 (8.1)	94.0 (8.3)	93.6 (7.7)	0.005	0.326
Young adults BMI, kg/m^2^	21.5 (3.7)	21.9 (3.9)	21.5 (3.5)	21.3 (3.7)	0.191	0.175	21.3 (3.9)	21.4 (3.4)	22.1 (4.0)	0.115	0.199	22.4 (4.3)	21.4 (3.5)	21.1 (3.4)	0.003	0.331
WHtR	0.5 (0.1)	0.5 (0.1)	0.4 (0.1)	0.4 (0.1)	0.060	0.166	0.4 (0.1)	0.4 (0.1)	0.5 (0.1)	0.036	0.272	0.5 (0.1)	0.4 (0.1)	0.4 (0.1)	0.012	0.321
Smoking history					0.353	0.051				0.756	0.031				0.021	0.117
Yes	275 (37.1)	104 (40.6)	79 (35.0)	92 (35.5)			93 (35.4)	102 (37.8)	80 (38.5)			102 (44.3)	96 (34.9)	77 (32.6)		
No	466 (62.9)	152 (59.4)	147 (65.0)	167 (64.5)			170 (64.6)	168 (62.2)	128 (61.5)			128 (55.7)	179 (65.1)	159 (67.4)		
Young adults SBP, mmHg	114.6 (12.9)	115.4 (12.6)	113.4 (12.8)	114.8 (13.3)	0.295	0.046	114.0 (13.2)	115.1 (12.1)	114.6 (13.7)	0.675	0.045	116.6 (13.5)	114.5 (13.1)	113.0 (12.0)	0.028	0.282
Young adults DBP, mmHg	75.0 (10.6)	75.0 (10.3)	74.2 (10.6)	75.6 (10.9)	0.430	0.054	75.1 (10.4)	75.0 (10.6)	74.9 (11.1)	0.987	0.017	75.7 (11.1)	74.7 (10.1)	74.7 (10.7)	0.589	0.095
Daily energy intake, kcal	1028.9 (493.8)	834.2 (338.0)	917.1 (441.0)	1169.9 (530.7)	<0.001	0.755	1100.0 (537.7)	969.6 (430.5)	977.2 (480.4)	0.017	0.241	1158.2 (590.6)	1006.3 (494.8)	932.0 (356.9)	<0.001	0.464
DDS	5.5 (2.1)	3.1 (0.8)	5.5 (0.5)	7.8 (0.8)	<0.001	6.023	6.5 (1.9)	5.2 (2.0)	4.6 (1.9)	<0.001	0.956	5.4 (2.1)	5.0 (2.1)	6.1 (1.9)	<0.001	0.368
Healthful plant-based diet index	54.3 (6.8)	56.8 (5.6)	54.8 (6.7)	51.4 (7.0)	<0.001	0.846	46.9 (3.3)	55.3 (2.0)	62.4 (3.0)	<0.001	4.938	52.2 (7.2)	54.9 (6.2)	55.8 (6.5)	<0.001	0.522
EAT-Lancet score	10.0 (1.2)	9.8 (0.9)	10.1 (1.3)	10.1 (1.3)	0.003	0.304	9.8 (1.2)	10.0 (1.2)	10.2 (1.1)	<0.001	0.375	8.6 (0.7)	10.0 (0.0)	11.4 (0.6)	<0.001	4.234

^1^Obtained from ANOVA or Chi-square test, where appropriate.

SD, standard deviation; ASD, absolute standardized difference; BMI, body mass index; WHtR, waist-to-height ratio; SBP, systolic blood pressure; DBP, diastolic blood pressure; DDS, Dietary Diversity Score; h-PDI, healthful Plant-based Diet Index.

Values are mean (SD) for continuous variables and *n* (%) for categorical variables. *p*-values were calculated using Pearson chi-squared test for categorical variables and one-way ANOVA for continuous variables. ASD was calculated between T1 (lowest) and T3 (highest) tertiles. ASD < 0.1 indicates negligible imbalance between groups.

T1/T2/T3: tertiles of each dietary index score. Low, Medium, and High denote the first, second, and third tertiles, respectively.

Total *N =* 741 participants with complete dietary and outcome data. Some variables have missing values; N for each variable may differ slightly from the total.

Participants with higher DDS had younger parents, higher parental education, non-farming parental occupations, and recent physical activity engagement (all *p* < 0.05). For h-PDI, only paternal education differed significantly (*p* = 0.007). Higher EAT-Lancet scores were more common among females and non-smokers, also differed by school enrollment status. Absolute standardized differences (ASD) between the lowest and highest tertiles are also reported, allowing readers to assess the practical magnitude of between-group differences beyond statistical significance; ASD < 0.1 indicates negligible imbalance.

### 3.2 Health outcomes in the overall sample and by gender

Table 4 presents metabolic outcomes for the overall sample and stratified by gender. Among all participants, 29 (5.1%) met the criteria for MetS, 217 (38.2%) had at least one metabolic problem, 122 (21.1%) were classified as overweight or obese, 257 (44.7%) had elevated BP or hypertension, and 103 (17.8%) had a high WHtR.

**Table 4:** Overall and gender-stratified metabolic outcomes by levels of dietary index *N* (%).

Metabolism outcomes	Total	DDS				h-PDI				Eat-Lancet			
		Low	Medium	High	*p-*value^1^	Low	Medium	High	*p-*value^1^	Low	Medium	High	*p-*value^1^
MetS													
No	536 (94.87)	173 (93.01)	166 (95.95)	197 (95.63)	0.371	203 (93.98)	205 (96.24)	128 (94.12)	0.513	152 (91.02)	194 (95.57)	190 (97.44)	0.019
Yes	29 (5.13)	13 (6.99)	7 (4.05)	9 (4.37)		13 (6.02)	8 (3.76)	8 (5.88)		15 (8.98)	9 (4.43)	5 (2.56)	
Metabolic problems													
No	351 (61.80)	111 (59.36)	116 (67.05)	124 (59.62)	0.233	128 (58.99)	143 (66.82)	80 (58.39)	0.158	93 (55.69)	129 (63.55)	129 (65.15)	0.146
Yes	217 (38.20)	76 (40.64)	57 (32.95)	84 (40.38)		89 (41.01)	71 (33.18)	57 (41.61)		74 (44.31)	74 (36.45)	69 (34.85)	
Overweight or obesity													
Normal	457 (78.93)	148 (77.08)	140 (80.00)	169 (79.72)	0.439	177 (79.73)	173 (80.09)	107 (75.89)	0.629	119 (70.83)	170 (80.57)	168 (84.00)	0.007
Overweight/obesity	122 (21.07)	44 (22.92)	35 (20.00)	43 (20.28)		45 (20.27)	43 (19.91)	34 (24.11)		49 (29.17)	41 (19.43)	32 (16.00)	
Blood pressure													
Normal	318 (55.30)	106 (55.79)	103 (58.86)	109 (51.90)	0.517	120 (54.30)	120 (55.81)	78 (56.12)	0.902	84 (50.00)	117 (55.71)	117 (59.39)	0.202
Elevated BP/hypertension	257 (44.70)	84 (44.21)	72 (41.14)	101 (48.10)		101 (45.70)	95 (44.19)	61 (43.88)		84 (50.00)	93 (44.29)	80 (40.61)	
WHtR													
Normal	476 (82.21)	149 (77.60)	148 (84.57)	179 (84.43)	0.124	188 (84.68)	180 (83.33)	108 (76.60)	0.125	130 (77.38)	171 (81.04)	175 (87.50)	0.035
Too high	103 (17.79)	43 (22.40)	27 (15.43)	33 (15.57)		34 (15.32)	36 (16.67)	33 (23.40)		38 (22.62)	40 (18.96)	25 (12.50)	
stratified by gender^2^													
Male													
MetS													
No	263 (91.96)	80 (89.89)	78 (91.76)	105 (93.75)	0.605	105 (90.52)	94 (94.00)	64 (91.43)	0.633	93 (88.57)	89 (93.68)	81 (94.19)	0.274
Yes	23 (8.04)	9 (10.11)	7 (8.24)	7 (6.25)		11 (9.48)	6 (6.00)	6 (8.57)		12 (11.43)	6 (6.32)	5 (5.81)	
Metabolic problems													
No	146 (50.69)	46 (51.11)	45 (52.94)	55 (48.67)	0.834	56 (48.28)	55 (54.46)	35 (49.30)	0.638	50 (47.62)	50 (52.63)	46 (52.27)	0.731
Yes	142 (49.31)	44 (48.89)	40 (47.06)	58 (51.33)		60 (51.72)	46 (45.54)	36 (50.70)		55 (52.38)	45 (47.37)	42 (47.73)	
Overweight or obesity													
Normal	221 (75.17)	68 (73.12)	64 (74.42)	89 (77.39)	0.616	90 (76.92)	75 (72.82)	56 (75.68)	0.866	69 (65.09)	77 (78.57)	75 (83.33)	0.007
Overweight/obesity	73 (24.83)	25 (26.88)	22 (25.58)	26 (22.61)		27 (23.08)	28 (27.18)	18 (24.32)		37 (34.91)	21 (21.43)	15 (16.67)	
Blood pressure													
Normal	124 (42.61)	39 (42.86)	44 (51.16)	41 (35.96)	0.363	45 (38.46)	46 (45.10)	33 (45.83)	0.310	43 (40.57)	40 (41.24)	41 (46.59)	0.500
Elevated BP/hypertension	167 (57.39)	52 (57.14)	42 (48.84)	73 (64.04)		72 (61.54)	56 (54.90)	39 (54.17)		63 (59.43)	57 (58.76)	47 (53.41)	
WHtR													
Normal	222 (75.51)	66 (70.97)	63 (73.26)	93 (80.87)	0.217	90 (76.92)	78 (75.73)	54 (72.97)	0.824	76 (71.70)	71 (72.45)	75 (83.33)	0.116
Too high	72 (24.49)	27 (29.03)	23 (26.74)	22 (19.13)		27 (23.08)	25 (24.27)	20 (27.03)		30 (28.30)	27 (27.55)	15 (16.67)	
Female													
MetS													
No	273 (97.85)	93 (95.88)	88 (100.00)	92 (97.87)	0.155	98 (98.00)	111 (98.23)	64 (96.97)	0.847	59 (95.16)	105 (97.22)	109 (100.00)	0.094
Yes	6 (2.15)	4 (4.12)	0 (0.00)	2 (2.13)		2 (2.00)	2 (1.77)	2 (3.03)		3 (4.84)	3 (2.78)	0 (0.00)	
Metabolic problems													
No	205 (73.21)	65 (67.01)	71 (80.68)	69 (72.63)	0.110	72 (71.29)	88 (77.88)	45 (68.18)	0.317	43 (69.35)	79 (73.15)	83 (75.45)	0.686
Yes	75 (26.79)	32 (32.99)	17 (19.32)	26 (27.37)		29 (28.71)	25 (22.12)	21 (31.82)		19 (30.65)	29 (26.85)	27 (24.55)	
Overweight or obesity													
Normal	236 (82.81)	80 (80.81)	76 (85.39)	80 (82.47)	0.540	87 (82.86)	98 (86.73)	51 (76.12)	0.196	50 (80.65)	93 (82.30)	93 (84.55)	0.567
Overweight/obesity	49 (17.19)	19 (19.19)	13 (14.61)	17 (17.53)		18 (17.14)	15 (13.27)	16 (23.88)		12 (19.35)	20 (17.70)	17 (15.45)	
Blood pressure													
Normal	194 (68.31)	67 (67.68)	59 (66.29)	68 (70.83)	0.890	75 (72.12)	74 (65.49)	45 (67.16)	0.424	41 (66.13)	77 (68.14)	76 (69.72)	0.475
Elevated BP/hypertension	90 (31.69)	32 (32.32)	30 (33.71)	28 (29.17)		29 (27.88)	39 (34.51)	22 (32.84)		21 (33.87)	36 (31.86)	33 (30.28)	
WHtR													
Normal	254 (89.12)	83 (83.84)	85 (95.51)	86 (88.66)	0.037	98 (93.33)	102 (90.27)	54 (80.60)	0.029	54 (87.10)	100 (88.50)	100 (90.91)	0.715
Too high	31 (10.88)	16 (16.16)	4 (4.49)	11 (11.34)		7 (6.67)	11 (9.73)	13 (19.40)		8 (12.90)	13 (11.50)	10 (9.09)	

1 Obtained from Chi-square or trend Chi-square test, where appropriate.

2 The metabolic outcomes showed significant differences between men and women. (MetS: *p* = 0.002; Having metabolic problem: *p* < 0.001; Overweight or Obesity: *p* = 0.024; Blood pressure: *p* < 0.001; WHtR: *P* < 0.001) (Supplementary Table S1).

MetS, metabolic syndrome; BP, blood pressure; WHtR, waist-to-height ratio; DDS, Dietary Diversity Score; h-PDI, healthful Plant-based Diet Index.

Values are *n* (%). *p*-values were calculated using Pearson chi-squared test. Low, Medium, and High denote the first, second, and third tertiles of each dietary index score, respectively.

Gender-stratified results are presented separately for males and females. Total *N =* 741.

Males had significantly higher prevalence rates than females across all outcomes: MetS (8.0% vs. 2.2%, *p* = 0.002), at least one metabolic problem (49.3% vs. 26.8%, *p* < 0.001), overweight/obesity (24.8% vs. 17.2%, *p* = 0.024), elevated BP/hypertension (57.4% vs. 31.7%, *p* < 0.001), and high WHtR (24.5% vs. 10.9%, *p* < 0.001). No significant gender × dietary index or trajectory interactions were observed (*P* for interaction > 0.05 for all outcomes; Supplementary Table S7).

### 3.3 Current dietary indices and health outcomes

After adjustment, higher DDS was inversely associated with overweight/obesity (T3 vs. T1: OR 0.262, 95% CI 0.099–0.691, *p* = 0.007, *P* for trend = 0.007) and high WHtR (T2 vs. T1: OR 0.291, 95% CI 0.105–0.808, *p* = 0.018; T3 vs. T1: OR 0.259, 95% CI 0.092–0.728, *p* = 0.010, *P* for trend = 0.010) (Table 1). DDS showed no significant associations with MetS, metabolic problems, or elevated BP. h-PDI was positively associated with high WHtR in the highest tertile (T3 vs. T1: OR 3.005, 95% CI 1.108–8.152, *p* = 0.031, *P* for trend = 0.030) but was not associated with MetS, metabolic problems, overweight/obesity, or elevated BP. The EAT-Lancet score was inversely associated with overweight/obesity (T2 vs. T1: OR 0.339, 95% CI 0.145–0.795, *p* = 0.013; T3 vs. T1: OR 0.340, 95% CI 0.148–0.778, *p* = 0.011, *P* for trend = 0.009). No significant associations were observed between the EAT-Lancet score and MetS (*p* = 0.519), metabolic problems (*p* = 0.372), elevated BP (*p* = 0.873), or high WHtR (*p* = 0.983). After FDR correction, four of the 15 cross-sectional comparisons retained statistical significance: DDS with overweight/obesity (*Q* = 0.016), EAT-Lancet score with overweight/obesity (*Q* = 0.016), DDS with high WHtR (*Q* = 0.031), and h-PDI with high WHtR (*Q* = 0.046) (Supplementary Table S8).

### 3.4 Four life stages dietary trajectories and metabolic outcomes

The trajectory analysis included 298 of the 741 participants with complete dietary data across all four stages. Supplementary Table S4 compares the baseline characteristics of these 298 participants against the remaining 1,215 members of the original birth cohort who were not included in the trajectory analysis (comprising both those lost to follow-up and those with incomplete dietary data); this full cohort served as the reference population for inverse probability weighting.

**Figure 2:**
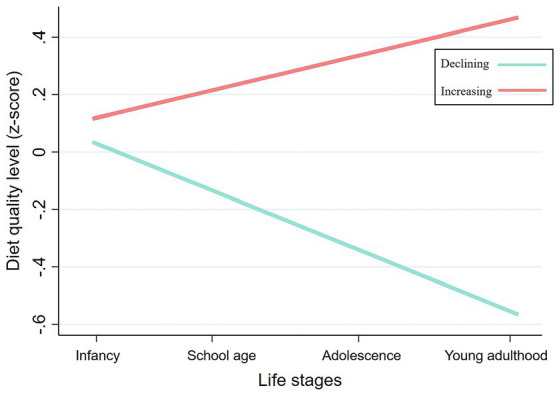
Trajectories of Dietary Quality across Four Life Stages. Group-based trajectory modeling identified two dietary quality trajectories among 298 participants with complete dietary data: an increasing trajectory (*n* = 158, 53.0%) and a declining trajectory (*n* = 140, 47.0%). The x-axis represents the four developmental stages (T1: infancy; T2: school age; T3: adolescence; T4: young adulthood), and the y-axis represents the standardized dietary quality score (z-score). At each stage, the dietary index most appropriate for that period was used: the infant and young child feeding (IYCF) index in infancy and the Dietary Diversity Score (DDS) at the three later stages, both converted to z-scores for comparability. The two groups started at similar dietary quality levels in infancy (z-score ≈ 0.1) and diverged over time. The increasing group showed a steady rise in dietary quality across the four stages, while the declining group showed a gradual decrease.

Using GBTM, two dietary quality trajectories were identified (Figure 2): an increasing trajectory (*n* = 158, 53.0%) and a declining trajectory (*n* = 140, 47.0%). The increasing trajectory showed moderate dietary quality in infancy and childhood, with improvement in adolescence and young adulthood. The declining trajectory started with higher dietary quality in infancy that decreased over time. Model selection based on BIC considered solutions ranging from one to four groups. Despite the one-group model having the lowest BIC (−1358.52), the two-group model (BIC = −1357.48) was retained because it balanced parsimony with interpretability, yielding distinct trajectories that converged successfully. Attempts to fit three or more groups resulted in non-convergence due to singular variance matrices (Supplementary Table S6). The entropy value was 0.307, with a minimum average posterior probability of 0.492, indicating modest classification certainty.

In Figure 2, the x-axis represents the four developmental stages (T1: infancy; T2: school age; T3: adolescence; T4: young adulthood), and the y-axis represents *z*-score at each stage, with higher values indicating better dietary quality. The solid line (increasing trajectory) and dashed line (declining trajectory) represent the estimated mean *z*-scores for each group across the four stages.

Table 2 presents the associations between dietary trajectories and metabolic outcomes. Compared with the declining trajectory, the increasing trajectory was associated with lower odds of overweight/obesity (OR 0.34, 95% CI 0.15–0.77, *p* = 0.009). For high WHtR, the association was in the same direction but did not reach statistical significance (OR 0.44, 95% CI 0.18–1.06, *p* = 0.067). No significant associations were observed for MetS (OR 0.35, 95% CI 0.07–1.70, *p* = 0.195), metabolic problems (OR 1.24, 95% CI 0.67–2.30, *p* = 0.490), or elevated BP/hypertension (OR 0.99, 95% CI 0.53–1.85, *p* = 0.972). All baseline maternal and birth characteristics—including gender, treatment group, parental education and occupation, household wealth index, birth weight, gestational age, and parental age—were comparable between the 298 participants in the trajectory analysis and the 1,215 participants not included (Supplementary Table S4), with all absolute standardized differences below 0.1. Although systolic blood pressure (SBP), diastolic blood pressure (DBP), and the prevalence of elevated blood pressure differed significantly between groups, these are outcome variables measured at young adulthood and do not reflect selection bias at baseline. The comparability of early-life characteristics supports the representativeness of the trajectory subsample with respect to measured baseline factors.

### 3.5 Life-stage-specific analysis

To examine whether the observed life-course dietary effect reflects a continuous pattern or is driven by a specific critical period, dietary quality at each developmental stage was entered simultaneously as an independent exposure in a single regression model (Supplementary Table S10). Infant diet (IYCF), school-age DDS, and adolescent DDS showed no independent association with any metabolic outcome after adjusting for adult diet (all *p* > 0.1). In contrast, adult dietary quality was the primary driver: adult DDS was significantly associated with BMI (*β* = −1.71, *p* = 0.033) and WHtR (*β* = −0.025, *p* = 0.027). These findings support a critical period interpretation, with the dietary effect concentrated in young adulthood rather than distributed across the life course.

## 4 Discussion

This study compared three dietary indices and examined dietary quality trajectories across four life stages in a rural Chinese birth cohort with up to 20 years of follow-up. The EAT-Lancet score and DDS showed inverse associations with overweight/obesity, DDS was inversely associated with high WHtR, and h-PDI was positively associated with high WHtR. After FDR correction, four associations retained statistical significance: DDS with overweight/obesity (*Q* = 0.016), EAT-Lancet score with overweight/obesity (*Q* = 0.016), DDS with high WHtR (*Q* = 0.031), and h-PDI with high WHtR (*Q* = 0.046). The trajectory analysis identified two patterns: an increasing and a declining trajectory. The increasing trajectory was associated with lower odds of overweight/obesity (Table 2); the association with high WHtR did not reach statistical significance (*p* = 0.067). WHtR is not included in the Chinese diagnostic criteria for MetS (25) but is an important indicator in other standards and was therefore included (35, 36).

The inverse association between DDS and overweight/obesity in our study, which remained significant after FDR correction, is consistent with findings from Japan (37), Korea (38), Iran (39, 40). The absence of a significant association between DDS and MetS aligns with studies from Sri Lanka (41) and China (22, 42), although some studies in these settings reported null or positive associations between dietary diversity and adiposity, likely reflecting different dietary contexts. A study among European immigrants also found no significant relationship (43). These discrepancies may reflect differences in sample size, age range, dietary assessment methods, and DDS definitions.

Regarding h-PDI, some studies have found protective associations with metabolic outcomes (12, 44–46), while others have reported no significant association (47–49). The possible benefits of h-PDI may come from reducing animal-based foods. Frequent intake of such foods, especially red meat, has been linked to poor metabolic outcomes (50, 51). However, in China, foods like eggs, fish, and dairy are considered part of a healthy and balanced diet (52–55). A high h-PDI score may therefore indicate an overly restrictive or monotonous diet, which could relate to less favorable eating behaviors (56–58).

Relatively fewer studies have evaluated the EAT-Lancet score. Available evidence suggests that it may be associated with a lower risk of overweight and obesity (13, 40, 59). The EAT-Lancet score limits red meat intake but permits moderate consumption of other animal products, which may promote a more balanced diet. It also emphasizes reducing added sugar, a recognized risk factor for MetS (60, 61). Among the three indices, both the EAT-Lancet score and DDS showed significant inverse associations with overweight/obesity that survived FDR correction; the EAT-Lancet score showed a slightly stronger point estimate, though formal comparisons of discriminative ability revealed no significant differences. Receiver operating characteristic (ROC) analysis (Supplementary Table S9) showed that all three indices had similar discriminatory ability across outcomes (area under the curve [AUC] range 0.660–0.869), with no significant differences between indices (DeLong test *p* ≥ 0.186 for all outcomes). For overweight/obesity, AUC values were 0.748 (DDS), 0.724 (h-PDI), and 0.752 (EAT-Lancet score). These results do not support declaring any single index as superior. This suggests that although the EAT-Lancet score exhibited a statistically significant association, its actual predictive advantage over the other indices is minimal. Further studies with larger samples are warranted to confirm these associations and to formally compare predictive performance.

Dietary patterns change over time, and their influence on metabolic health may accumulate across the life course. We used GBTM to characterize dietary quality across four life stages. Two trajectory patterns were identified: increasing and declining. The increasing trajectory group maintained or improved their dietary quality over time and showed lower odds of overweight/obesity in young adulthood (OR 0.34, *p* = 0.009). The association with high WHtR was in the same direction but did not reach statistical significance (OR 0.44, *p* = 0.067). The association with MetS was also in the same direction (OR 0.35) but was not significant, likely because of the small number of MetS cases (*n* = 29). This observation is consistent with previous studies suggesting that long-term dietary patterns may be more informative than single time-point assessments (62, 63). The low entropy (0.307) of the two-group model suggests imperfect classification precision, which may have attenuated the observed associations.

The two trajectory groups started at similar dietary quality levels in infancy (*z-*score ≈ 0.1) and then diverged over time (Figure 2). The increasing group showed a steady rise in dietary quality across the four stages, while the declining group showed a gradual decrease. From these trajectories, dietary quality differences were smallest during school age and widened again in early adulthood. The lack of differentiation in infancy is likely because infant diets are limited to breast milk and a narrow range of complementary foods, resulting in relatively homogeneous feeding practices across households in this rural setting. During school age, government-provided school lunches improved diet quality among children from low-income households and reduced dietary disparities, making children’s patterns more similar. By early adolescence, school lunches were no longer provided, and caregivers remained the primary decision-makers for household food choices; dietary disparities began to widen again. In early adulthood, parental influence declined and individual lifestyle choices—such as leaving home, starting work, or greater autonomy in food choices—became more important, further widening the gap between the two groups. Individuals who maintained healthier dietary patterns throughout growth tended to have better metabolic outcomes in early adulthood.

To our knowledge, this is the first long-term longitudinal study in a rural Chinese population to evaluate dietary quality across multiple life stages using detailed dietary indices. Such an approach is more informative than constructing trajectories from a single index or relying on assessments at only one stage of life, as it better reflects the dynamic nature of diet during growth and development. The trajectories identified in this study likely provide an accurate picture of changes in dietary quality over the first 20 years of life among rural children in China, and offer important evidence for dietary management during this period.

Both the cross-sectional analyses—where DDS and EAT-Lancet score were inversely associated with overweight/obesity—and the trajectory analysis identified protective associations, suggesting that cumulative dietary exposure may influence metabolic risk. The trajectory approach captures dynamic dietary change from infancy to young adulthood, yet the magnitude of association with overweight/obesity was comparable between the trajectory (OR 0.34) and the EAT-Lancet score at a single time point (OR 0.34). This indicates that comprehensive single-point indices may perform similarly to longitudinal trajectories for certain outcomes. The trajectory analysis, however, revealed distinct patterns—improving versus declining quality—that cross-sectional data alone cannot capture, highlighting the value of repeated measurements for understanding dietary evolution. Although this study was not designed to formally compare predictive performance of trajectory versus cross-sectional models, the findings support integrating both long-term dietary monitoring and targeted single-point screening into metabolic health strategies. These results may inform early-life nutrition programs and school-based interventions for preventing metabolic problems. They align with global initiatives, including the WHO’s life-course approach to nutrition and the Sustainable Development Goals (64–66), which emphasize improving diets from early childhood through adulthood to reduce the burden of noncommunicable diseases and promote health equity worldwide.

This study has several limitations. First, it was conducted within a trial of antenatal micronutrient supplementation, which may limit generalizability. Second, only 298 of 4,604 original trial participants (6.5%) had complete dietary data for the trajectory analysis. A formal attrition analysis comparing baseline characteristics between the 298 included and 4,306 excluded individuals showed negligible differences in maternal age (SMD = 0.036), birth weight (SMD = 0.090), parental education (ASD ≤ 0.050), and household wealth (ASD = 0.047) (Supplementary Table S4). Although these findings support the MAR assumption underlying IPW, the possibility of MNAR bias cannot be excluded if dropout is systematically related to unmeasured factors such as socioeconomic instability or health literacy. Under MNAR conditions, IPW provides only partial correction, and the trajectory findings should be interpreted with caution. Third, different dietary indices were used across life stages (IYCF in infancy, DDS thereafter), which may limit comparability. Although *z-*score standardization was applied and sensitivity analyses excluding infancy yielded consistent results, this heterogeneity should be considered when interpreting the trajectories. Fourth, the simplified FFQ used in this study, while previously applied in similar rural Chinese populations, was not specifically validated against a gold-standard dietary assessment method, such as multiple 24-h recalls or weighed food records. Although meta-analytic evidence supports the suitability of FFQs for dietary intake assessment in nutritional epidemiological research (29), and a related validation study in a rural Chinese population demonstrated moderate to good reproducibility and validity of an FFQ with similar food items (30), the specific instrument used here has not undergone formal validation. This may introduce non-differential misclassification of dietary exposures, which would likely attenuate the observed associations toward the null. Consequently, the significant associations reported in this study may represent conservative estimates, although the possibility of systematic error cannot be excluded. Fifth, the sample size for the trajectory analysis was relatively small (*N* = 298). As detailed in Section 2.2 and Supplementary Table S11, post-hoc power analysis indicated adequate power for a primary outcome of overweight/obesity (power = 0.88) but insufficient power for rare outcomes such as MetS (power = 0.32), which may explain the wide confidence intervals observed for these outcomes (Table 2). The modest entropy (0.307) and minimal BIC difference between one-group and two-group models (ΔBIC = 1.04) are consistent with the concern that GBTM may identify groups in continuously distributed data (23). The trajectory groups should therefore be interpreted as statistical approximations for summarizing dietary patterns rather than as evidence of distinct subpopulations (24), and the trajectory findings should be considered hypothesis-generating rather than confirmatory. Sixth, although we applied stratified FDR correction for multiple comparisons across dietary indices within each metabolic outcome (Supplementary Table S8), the simultaneous evaluation of multiple metabolic sub-outcomes may still inflate the family-wise Type I error rate. These findings should therefore be interpreted as hypothesis-generating rather than confirmatory. Seventh, the study was conducted in rural China, and findings may not apply to populations with different dietary patterns. Finally, genetic factors were unavailable and the trajectory analyses did not adjust for energy intake; these unmeasured confounders may bias the estimated associations.

## 5 Conclusion

In this rural Chinese birth cohort with 20 years of follow-up, higher DDS was associated with lower odds of overweight/obesity and high WHtR, and the EAT-Lancet score was associated with lower odds of overweight/obesity after correction for multiple testing. Higher h-PDI was associated with higher odds of high WHtR. An increasing dietary quality trajectory from infancy to young adulthood was also associated with lower odds of overweight/obesity. All three dietary indices showed similar discriminative ability, suggesting that simpler tools like DDS may be equally useful for population-level dietary assessment. These findings support the importance of promoting healthy dietary habits throughout the life course and require confirmation in larger, more diverse populations.

## Data Availability

The raw data supporting the conclusions of this article will be made available by the authors, without undue reservation.
