# Supplementary material for: Dietary quality across the life course and metabolic syndrome: evidence from two decades of follow-up in rural China young adults
Source: Front Public Health. 2026 Jun 23;14:1826210. doi: 10.3389/fpubh.2026.1826210 (PMC13337929; doi:10.3389/fpubh.2026.1826210)
Supplement: Supplementary file 1 [file Data_Sheet_1.docx]

**Figure S1.** Directed acyclic graph (DAG) for the association between dietary measures and metabolic outcomes.

**
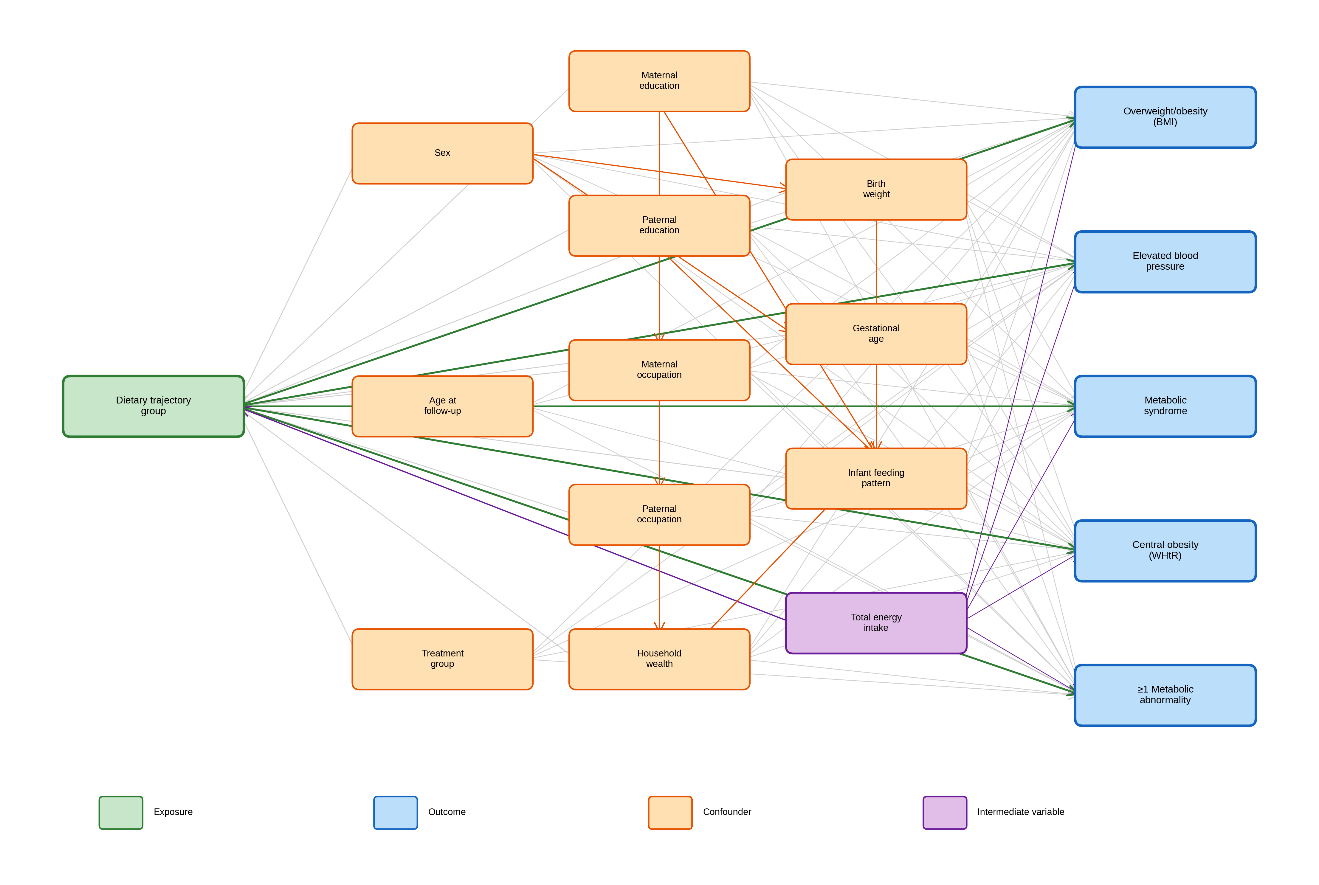
**

The DAG was constructed a priori to identify the minimally sufficient adjustment set. Green nodes represent the exposure (dietary trajectory group); blue nodes represent the outcomes (overweight/obesity, elevated blood pressure, metabolic syndrome, high waist-to-height ratio, and having at least one metabolic abnormality); orange nodes represent confounders (maternal education, paternal education, maternal occupation, paternal occupation, household wealth, sex, birth weight, gestational age, infant feeding pattern, treatment group, age, smoking history, physical activity, sleep quality, and employment status); purple nodes represent mediators (total energy intake). Arrows indicate hypothesized causal directions. The DAG informed the selection of covariates for the logistic regression models. Total energy intake was adjusted for in the dietary index analyses (Table 3) but not in the trajectory analyses (Table 4), as trajectory groups reflect dietary quality patterns rather than absolute intake.

**Table S1.** Overall And Gender-Stratified Metabolism Outcomes. N(%)

| Metabolism Outcomes | Total | Male | Female | *P-*value |
| --- | --- | --- | --- | --- |
|  |  |  |  |  |
| Metabolic syndrome |  |  |  |  |
| No | 536(94.87) | 263(91.96) | 273(97.85) | 0.002 |
| Yes | 29(5.13) | 23(8.04) | 6(2.15) |  |
| Metabolic problems |  |  |  |  |
| No | 351(61.80) | 146(50.69) | 205(73.21) | <0.001 |
| Yes | 217(38.20) | 142(49.31) | 75(26.79) |  |
| Overweight or Obesity |  |  |  |  |
| Normal | 457(78.93) | 221(75.17) | 236(82.81) | 0.024 |
| Overweight/Obesity | 122(21.07) | 73(24.83) | 49(17.19) |  |
| Blood pressure |  |  |  |  |
| Normal | 318(55.30) | 124(42.61) | 194(68.31) | <0.001 |
| Elevated BP/Hypertension | 257(44.70) | 167(57.39) | 90(31.69) |  |
| WHtR |  |  |  |  |
| Normal | 476(82.21) | 222(75.51) | 254(89.12) | <0.001 |
| Too high | 103(17.79) | 72(24.49) | 31(10.88) |  |

Abbreviations: WHtR, waist-to-height ratio; BP, blood pressure.

Values are n (%). P values were calculated using Pearson chi-squared test.

Total N = 741 participants with complete outcome data.

**Table S2.** Sensitivity analysis: associations between dietary trajectories and metabolic outcomes with inverse probability weighting

| Outcome | AOR | 95% CI | *P* |
| --- | --- | --- | --- |
| MetS | 0.009 | (0.001, 14.491) | 0.211 |
| Metabolic problems | 0.802 | (0.348, 1.848) | 0.604 |
| Overweight or Obesity | 0.094 | (0.028, 0.311) | <0.001 |
| Elevated BP/Hypertension | 0.684 | (0.267, 1.750) | 0.428 |
| WHtR | 0.223 | (0.075, 0.662) | 0.007 |

Abbreviations: AOR, adjusted odds ratio; CI, confidence interval; BP, blood pressure; MetS, metabolic syndrome; WHtR, waist-to-height ratio; IPW, inverse probability weighting.

Reference group: Declining trajectory. AORs were estimated using logistic regression with stabilized IPW weights. IPW weights were constructed using logistic regression with sex, treatment group, parental education, and parental occupation as predictors. Weights were stabilized and truncated at the 1st and 99th percentiles.

All models were adjusted for parental occupation, parental education, household wealth, treatment group, birth weight, moderate physical activity, sleep quality, sex, smoking history, employment status, and age.The MetS model showed complete separation for several covariates, resulting in an extremely wide confidence interval; this result should be interpreted with caution.

**Table S3.** Intake of food components by Dietary Index Category.

| **Diet indices** | **Food group(g/day)** | **Included foods** | **Total** | **Low** | **Medium** | **High** | ***P*^1^** |
| --- | --- | --- | --- | --- | --- | --- | --- |
| DDS | Cereals | rice, noodles, corn, sweets and desserts | 249.77(235.95) | 210.35(207.68) | 244.02(205.54) | 292.09(275.11) | <0.001 |
|  | Vegetables | all vegetables | 122.68(103.35) | 68.37(65.77) | 126.8(93.07) | 170.49(114.98) | <0.001 |
|  | Fruits | all fruits | 276.8(349.86) | 111.86(211.59) | 255.5(320.47) | 444.51(395.45) | <0.001 |
|  | Meat | beef, lamb, pork and chicken | 47.27(70.03) | 16.8(34.31) | 44.83(67.17) | 77.87(83.28) | <0.001 |
|  | Fish | fish, shrimp and crab | 8.89(17.91) | 2.73(9.83) | 4.94(12.20) | 17.7(23.24) | <0.001 |
|  | Eggs | eggs and processed egg products | 23.25(28.55) | 9.97(15.36) | 22.95(24.99) | 35.93(34.45) | <0.001 |
|  | Beans | dry beans and soy foods | 25.46(37.42) | 10.41(19.45) | 22.67(30.82) | 41.99(47.36) | <0.001 |
|  | Milk | all dairy foods | 84.25(123.17) | 34.55(94.81) | 59.68(75.81) | 150.39(146.37) | <0.001 |
|  | Oil | vegetable oil and animal oil | 15.14(25.72) | 3.83(7.50) | 10.35(16.08) | 29.68(34.8) | <0.001 |
| h-PDI | Nuts | peanuts and all melon seeds | 5.02(11.10) | 6.01(11.66) | 4.58(10.73) | 4.43(10.85) | 0.261 |
|  | Tea and coffee | tea and coffee | 0.18(0.31) | 0.23(0.35) | 0.14(0.29) | 0.15(0.29) | 0.013 |
|  | Potatoes | potatoes and processed potato products | 16.15(26.80) | 21.61(24.51) | 16.93(28.22) | 8.96(26.02) | <0.001 |
|  | Fruit juice | all fruit juice | 37.55(109.52) | 69.27(133.34) | 24.13(74.29) | 17.67(107.82) | <0.001 |
|  | Miscellaneous animal-derived foods | pizza and cream soup | 0.07(0.19) | 0.15(0.27) | 0.05(0.15) | 0.01(0.06) | <0.001 |
|  | Fruits | all fruits | 215.71(300.01) | 246.74(270.98) | 208.49(315.21) | 189.05(310.56) | 0.500 |
|  | Vegetables | all vegetables | 122.78(110.52) | 133(111.04) | 117.07(104.92) | 118.06(116.13) | 0.104 |
|  | Legumes and vegetarian protein alternatives | dry beans and soy foods | 24.02(37.26) | 25.38(38.14) | 21.76(35.21) | 25.23(38.69) | 0.743 |
|  | Whole grains | whole grain cereal, dark bread, brown rice, other grains | 17.36(37.16) | 13.73(24.75) | 16.6(37.64) | 22.44(46.76) | 0.201 |
|  | Refined grains | rice, white bread, noodles | 229.78(230.37) | 268.07(238.49) | 235.08(242.63) | 179.55(194.55) | <0.001 |
|  | Sugar-sweetened beverages | sugary colas, milk teas, soft drinks | 109.59(179.77) | 173.84(217.79) | 106.56(164.35) | 39.85(110.3) | <0.001 |
|  | Sweets and desserts | biscuits, cakes, chocolate | 4.73(12.26) | 7.99(12.10) | 4.61(15.32) | 1.15(5.34) | <0.001 |
|  | Dairy | all dairy foods | 78.85(119.38) | 117.36(135.92) | 71.14(121.00) | 44.25(78.07) | <0.001 |
|  | Eggs | eggs and processed egg products | 22.34(27.74) | 28.36(25.38) | 22.01(31.96) | 15.88(22.98) | <0.001 |
|  | Fish or seafood | fish, shrimp and crab | 5.25(14.76) | 8.88(17.07) | 4.11(13.34) | 2.49(12.63) | <0.001 |
|  | Meat | beef, lamb, pork and chicken | 40.71(60.38) | 64.6(69.77) | 34.84(59.24) | 20.58(36.64) | <0.001 |
|  | Animal fat | all fat meat | 3.29(14.44) | 6.81(22.29) | 2.1(8.60) | 0.71(4.75) | <0.001 |
| EAT-Lancet | Whole grains | rice, wheat, corn, and other | 247.07(237.62) | 313.75(325.03) | 209.52(199.23) | 222.58(133.37) | <0.001 |
|  |  | whole grain fiber | 6.09(5.41) | 6.61(6.74) | 5.04(4.88) | 6.79(3.72) | 0.013 |
|  | Tubers and starchy vegetables | potatoes, sweet potatoes and processed potatoes products | 20.35(35.29) | 25.64(51.66) | 14.86(23.29) | 21.5(22.1) | 0.136 |
|  | Vegetables | all vegetables | 119.97(106.31) | 111.04(109.61) | 96.84(89.91) | 160.33(110.81) | <0.001 |
|  | Fruits | all fruits | 224.5(305.29) | 198.66(346.73) | 178.25(250.22) | 314.23(299.94) | <0.001 |
|  | Dairy foods | all dairy foods | 72.82(115.00) | 96.94(156.40) | 51.54(80.69) | 72.4(87.96) | <0.001 |
|  | Protein sources | beef, lamb, pork | 30.62(48.38) | 46.94(63.03) | 21.94(40.63) | 23.29(30.57) | <0.001 |
|  |  | chicken | 14.68(28.37) | 23.02(43.08) | 9.74(16.19) | 11.4(13.08) | <0.001 |
|  |  | eggs and processed egg products | 22.24(28.30) | 29.99(36.09) | 17.26(22.07) | 19.77(22.97) | <0.001 |
|  |  | fish, shrimp and crab | 5.68(15.15) | 8.52(22.39) | 3.51(8.52) | 5.18(10.09) | 0.029 |
|  | Legumes | dry beans | 12.65(24.41) | 11.86(28.66) | 10.74(22.62) | 16.07(20.54) | 0.748 |
|  |  | soy foods | 11.72(20.96) | 14.92(28.44) | 9.2(17.41) | 11.15(12.51) | 0.091 |
|  |  | peanuts and all melon seeds | 5.3(12.82) | 4.14(13.52) | 3.86(9.40) | 8.53(15.1) | <0.001 |
|  | Added sugars | all added sugars | 18.56(24.77) | 25.06(35.57) | 15.46(17.38) | 15.28(14.83) | <0.001 |

^1^Differences across the Dietary Index Category were tested using generalized linear models. Abbreviations: DDS, Dietary Diversity Score; hPDI, healthy Plant-based Diet Index; SD, standard deviation.

Values are mean (SD) g/day unless otherwise specified. Differences across dietary index tertiles were tested using generalized linear models. *P* < 0.05 indicates a statistically significant difference. Low, Medium, and High denote the first, second, and third tertiles of each dietary index score, respectively. Total *N* = 741. Some food groups have missing values; N for each item may differ slightly.

**Table S4.** Baseline characteristics of included versus excluded participants from the original parent trial (N = 4,604)

| Variable | Excluded (n = 4,306) | Included (n = 298) | *P*-value | SMD/ASD |
| --- | --- | --- | --- | --- |
| Maternal age, mean (SD) | 25.7 (4.3) | 25.8 (4.6) | 0.551 | 0.036 |
| Birth weight (g), mean (SD) | 3,176 (437) | 3,215 (394) | 0.138 | 0.090 |
| Infant sex |  |  | 0.263 | 0.141 |
| Male | 44.2% | 58.4% |  |  |
| Female | 36.1% | 41.6% |  |  |
| Mother’s education |  |  | 0.305 | 0.049 |
| <3 years | 5.9% | 6.4% |  |  |
| Primary school | 26.3% | 31.2% |  |  |
| Junior high school | 52.4% | 48.0% |  |  |
| Senior high school and above | 13.9% | 14.1% |  |  |
| Father’s education |  |  | 0.472 | 0.050 |
| <3 years | 1.3% | 0.7% |  |  |
| Primary school | 13.7% | 12.8% |  |  |
| Junior high school | 60.5% | 65.4% |  |  |
| Senior high school and above | 23.1% | 21.1% |  |  |
| Household wealth index |  |  | 0.179 | 0.047 |
| Poorest (1st tertile) | 32.7% | 36.6% |  |  |
| 2nd tertile | 33.8% | 35.6% |  |  |
| Richest (3rd tertile) | 32.6% | 27.9% |  |  |

Abbreviations: SMD, standardized mean difference; ASD, absolute standardized difference.

SMD/ASD < 0.1 indicates a negligible difference between groups. *P*-values were derived from independent t-tests for continuous variables and chi-square tests for categorical variables.

Maternal and paternal education were categorized as: <3 years, primary school, junior high school, and senior high school and above. Household wealth index was derived from principal component analysis of household assets and divided into tertiles.

Infant sex percentages in the excluded group do not sum to 100% due to missing sex data (19.7% missing among excluded participants). The ASD for infant sex (0.141) is largely attributable to this differential missingness rather than a true compositional difference between included and excluded participants.

**Table S5**. Covariate Balance Before and After Inverse Probability Weighting

| Covariate | SMD Unweighted | SMD Weighted |
| --- | --- | --- |
| Gender | 0.009 | <0.001 |
| Birth weight | 0.071 | 0.016 |
| Age | 0.060 | 0.007 |
| Prenatal supplementation group | 0.016 | 0.005 |
| Maternal educational attainment | 0.065 | 0.004 |
| Paternal educational attainment | 0.018 | 0.006 |
| Maternal occupation | 0.007 | 0.003 |
| Paternal occupation | 0.025 | 0.001 |

Abbreviations: SMD, standardized mean difference.

SMD was calculated as the absolute difference in means divided by the pooled standard deviation for continuous variables, and as the maximum absolute proportion difference across categories for categorical variables.

Propensity scores were estimated using logistic regression with all covariates listed above. Stabilized IPW weights were truncated at the 1st and 99th percentiles.

Effective sample size after IPW: 282.3 (original *N*=298).

All SMD values were <0.1 both before and after weighting, indicating adequate covariate balance.

**Table S6**. Model Selection for Group-Based Trajectory Analysis

| Number of Groups | BIC | AIC | Log-likelihood | Entropy | Min Avg Posterior Prob | Convergence |
| --- | --- | --- | --- | --- | --- | --- |
| 1 | -1358.52 | -1352.97 | -1349.97 | — | — | OK |
| 2 | -1357.48 | -1346.39 | -1340.39 | 0.307 | 0.492 | OK |
| 3 | -1366.03 | -1349.39 | -1340.39 | 0.269 | 0.179 | Singular variance matrix |
| 4 | -1374.57 | -1352.39 | -1340.39 | 0.190 | 0.168 | Singular variance matrix |

Abbreviations: BIC, Bayesian Information Criterion; AIC, Akaike Information Criterion.

Models were fitted using group-based trajectory modeling (GBTM) with a censored normal distribution. *N* = 298 refers to the trajectory analysis sample with complete data across all time points.The 2-group model was selected based on (1) convergence, (2) BIC, and (3) substantive interpretability. The 3- and 4-group models failed to converge due to a singular variance-covariance matrix.The 3-group model showed near-identical parameter estimates for Groups 2 and 3, indicating a false split rather than a meaningful third trajectory. Entropy for the 2-group model (0.307) is acceptable given the small sample size and limited number of time points.

**Table S7**. P Values for Sex × Dietary Index/Trajectory Interaction Terms

|  | Metabolic problems | Overweight or Obesity | Elevated BP/Hypertension | WHtR | MetS |
| --- | --- | --- | --- | --- | --- |
| DDS | 0.363 | 0.613 | 0.240 | 0.085 | 0.403^1^ |
| hPDI | 0.839 | 0.452 | 0.288 | 0.213 | 0.569 |
| EAT-Lancet | 0.883 | 0.501 | 0.859 | 0.696 | 0.466^1^ |
| Trajectory | 0.273 | 0.447 | 0.337 | 0.060 | —^2^ |

Abbreviations: BP, blood pressure; WHtR, waist-to-height ratio; MetS, metabolic syndrome; DDS, Dietary Diversity Score; hPDI, healthy Plant-based Diet Index.

Values represent *P* values for the interaction term between sex (male/female) and each dietary index or trajectory group in logistic regression models.No significant sex interactions were identified (all *P* > 0.05), supporting the presentation of pooled (sex-combined) results in the main analysis.

^1^ P value from likelihood ratio test with 1 degree of freedom due to complete separation of one interaction term.

^2^ Interaction term could not be estimated due to complete separation in the trajectory × MetS model. **Table S8**. False Discovery Rate Correction for Multiple Comparisons

| Dietary Index | Outcome | Raw *P* | FDR *Q* |
| --- | --- | --- | --- |
| DDS | MetS | 0.145 | 0.434 |
|  | Metabolic problems | 0.402 | 0.533 |
|  | Overweight or Obesity | 0.007 | 0.016 |
|  | Elevated BP/Hypertension | 0.796 | 0.873 |
|  | WHtR | 0.01 | 0.031 |
| h-PDI | MetS | 0.961 | 0.961 |
|  | Metabolic problems | 0.533 | 0.533 |
|  | Overweight or Obesity | 0.538 | 0.538 |
|  | Elevated BP/Hypertension | 0.506 | 0.873 |
|  | WHtR | 0.031 | 0.046 |
| EAT-Lancet | MetS | 0.519 | 0.778 |
|  | Metabolic problems | 0.372 | 0.533 |
|  | Overweight or Obesity | 0.011 | 0.016 |
|  | Elevated BP/Hypertension | 0.873 | 0.873 |
|  | WHtR | 0.984 | 0.984 |

Abbreviations: FDR, false discovery rate; BP, blood pressure; WHtR, waist-to-height ratio; MetS, metabolic syndrome; DDS, Dietary Diversity Score; hPDI, healthy Plant-based Diet Index.

FDR correction was applied using the Benjamini-Hochberg procedure, stratified by outcome (3 dietary indices per stratum). Only Tertile 3 vs Tertile 1 comparisons are shown.MetS used Firth penalized logistic regression.Four associations remained significant after FDR correction: DDS × overweight/obesity (Q = 0.016), EAT-Lancet × overweight/obesity (Q = 0.016), DDS × elevated WHtR (Q = 0.031), and h-PDI × elevated WHtR (Q = 0.046).All models adjusted for parental occupation, parental education, household wealth, treatment group, birth weight, moderate physical activity, sleep quality, sex, smoking history, employment status, age, and energy intake.

**Table S9**. Receiver operating characteristic (ROC) curve analysis: discriminatory ability of three dietary index trajectories for predicting metabolic outcomes in early adulthood

| Outcomes | DDS | | h-PDI | | EAT-Lancet | | DeLong test |
| --- | --- | --- | --- | --- | --- | --- | --- |
|  | AUC | 95% CI | AUC | 95% CI | AUC | 95% CI |  |
| MetS | 0.8694 | 0.775-0.964 | 0.8677 | 0.782-0.954 | 0.865 | 0.771-0.959 | *χ*²=0.14, *P*=0.931 |
| Metabolic problems | 0.671 | 0.604-0.738 | 0.6716 | 0.603-0.740 | 0.6603 | 0.592-0.729 | *χ*²=0.82, *P*=0.664 |
| Overweight or Obesity | 0.7482 | 0.682-0.814 | 0.7241 | 0.655-0.793 | 0.7523 | 0.684-0.821 | *χ*²=3.36, *P*=0.186 |
| Elevated BP/Hypertension | 0.7018 | 0.637-0.767 | 0.7032 | 0.639-0.768 | 0.7028 | 0.638-0.768 | *χ*²=0.08, *P*=0.959 |
| WHtR | 0.7986 | 0.727-0.870 | 0.7851 | 0.717-0.854 | 0.7717 | 0.699-0.845 | *χ*²=2.84, *P*=0.242 |

Abbreviations: MetS, metabolic syndrome; BP, blood pressure; WHtR, waist-to-height ratio.AUC, area under the ROC curve; CI, confidence interval.

ROC curves were derived from logistic regression models predicting each metabolic outcome from dietary index trajectories (categorical), adjusted for parental occupation, parental educational attainment, smoking history, sleep quality, gender, exercise in the past 7 days, school enrollment status, young adult age. DeLong test was used to compare AUCs across the three dietary indices within each outcome.All models were restricted to participants with complete data on all three dietary indices.

**Table S10**. Life-stage-specific analysis: independent effects of dietary quality at each developmental stage on continuous metabolic outcomes

| Outcome | *N* | Infant diet (IYCF) *β* (95% CI) | *P* | School-age diet (DDS) *β* (95% CI) | *P* | Adolescent diet (DDS) *β* (95% CI) | *P* | Adult diet *β* (95% CI) | *P* |
| --- | --- | --- | --- | --- | --- | --- | --- | --- | --- |
| *Adult diet = DDS* | | | | | | | | | |
| BMI (kg/m²) | 58 | 0.691 (-0.833-2.215) | 0.362 | -0.277(-1.228-0.675) | 0.558 | 0.760(-0.428-1.948) | 0.202 | -1.706(-3.261--0.151) | 0.033 |
| WHtR | 58 | 0.009(-0.013-0.030) | 0.434 | 0.005(-0.009-0.018) | 0.478 | 0.007(-0.010-0.024) | 0.385 | -0.025(-0.048--0.003) | 0.027 |
| SBP(mmHg) | 58 | 4.840(-0.634-10.314) | 0.081 | 0.057(-3.362-3.475) | 0.973 | 1.326(-2.942-5.595) | 0.531 | 0.131(-5.454-5.716) | 0.962 |
| DBP(mmHg) | 58 | 1.857(-3.588-7.301) | 0.492 | -0.319(-3.719-3.081) | 0.850 | -0.128(-4.373-4.117) | 0.951 | 1.556(-3.998-7.111) | 0.572 |
| TC(mmol/L) | 58 | 0.966(-2.832-4.764) | 0.608 | 0.896(-1.476-3.268) | 0.447 | 0.707(-2.255-3.668) | 0.630 | -3.261(-7.136-0.613) | 0.096 |
| TG(mmol/L) | 58 | -0.043(-0.330-0.245) | 0.765 | -0.040(-0.220-0.139) | 0.651 | -0.063(-0.287-0.161) | 0.571 | -0.264(-0.557-0.029) | 0.076 |
| LDL-C(mmol/L) | 58 | 0.008(-0.118-0.134) | 0.897 | -0.044(-0.123-0.035) | 0.264 | 0.000(-0.098-0.099) | 0.996 | -0.015(-0.144-0.113) | 0.807 |
| HDL-C(mmol/L) | 58 | -0.136(-0.366-0.093) | 0.236 | 0.037(-0.107-0.180) | 0.605 | -0.062(-0.241-0.117) | 0.483 | -0.035(-0.270-0.199) | 0.760 |
| *Adultdiet=h-PDI* | | | | | | | | | |
| BMI(kg/m²) | 49 | 1.595(-0.292-3.482) | 0.094 | -0.413(-1.533-0.707) | 0.454 | 0.865(-0.705-2.435) | 0.267 | 0.335(-1.051-1.722) | 0.623 |
| WHtR | 49 | 0.015(-0.010-0.041) | 0.223 | 0.011(-0.004-0.026) | 0.161 | 0.001(-0.021-0.022) | 0.952 | -0.005(-0.023-0.014) | 0.617 |
| SBP(mmHg) | 49 | 9.914(3.952-15.876) | 0.002 | -1.407(-4.947-2.132) | 0.421 | 4.503(-0.458-9.464) | 0.073 | 5.383(1.002-9.765) | 0.018 |
| DBP(mmHg) | 49 | 4.818(-1.637-11.274) | 0.137 | -1.594(-5.426-2.239) | 0.400 | 1.963(-3.409-7.335) | 0.459 | 2.120(-2.624-6.864) | 0.366 |
| TC(mmol/L) | 49 | 1.735(-2.652-6.122) | 0.423 | 1.665(-0.940-4.269) | 0.200 | -0.511(-4.162-3.139) | 0.775 | -1.389(-4.613-1.835) | 0.383 |
| TG(mmol/L) | 49 | 0.179(-0.144-0.503) | 0.264 | -0.038(-0.230-0.154) | 0.689 | -0.046(-0.315-0.223) | 0.727 | 0.150(-0.088-0.387) | 0.207 |
| LDL-C(mmol/L) | 49 | -0.007(-0.168-0.155) | 0.931 | -0.016(-0.112-0.080) | 0.734 | -0.021(-0.155-0.113) | 0.750 | -0.024(-0.143-0.095) | 0.681 |
| HDL-C(mmol/L) | 49 | -0.154(-0.423-0.116) | 0.252 | -0.007(-0.167-0.153) | 0.933 | -0.018(-0.242-0.207) | 0.871 | -0.049(-0.247-0.149) | 0.614 |
| *Adultdiet=EAT-Lancet* | | | | | | | | | |
| BMI(kg/m²) | 58 | 1.017(-0.521-2.555) | 0.188 | -0.088(-1.073-0.896) | 0.856 | 0.354(-0.875-1.583) | 0.561 | -0.874(-2.064-0.316) | 0.144 |
| WHtR | 58 | 0.013(-0.009-0.035) | 0.231 | 0.008(-0.006-0.022) | 0.265 | 0.001(-0.016-0.018) | 0.917 | -0.015(-0.032-0.001) | 0.070 |
| SBP(mmHg) | 58 | 4.872(-0.436-10.181) | 0.071 | 0.009(-3.388-3.407) | 0.996 | 1.445(-2.796-5.687) | 0.493 | 0.577(-3.529-4.682) | 0.777 |
| DBP(mmHg) | 58 | 1.742(-3.452-6.935) | 0.499 | -0.595(-3.919-2.728) | 0.718 | 0.522(-3.627-4.670) | 0.800 | 2.412(-1.604-6.428) | 0.230 |
| TC(mmol/L) | 58 | 1.467(-2.167-5.102) | 0.417 | 1.325(-1.001-3.652) | 0.254 | -0.256(-3.159-2.648) | 0.859 | -2.749(-5.560-0.062) | 0.055 |
| TG(mmol/L) | 58 | 0.035(-0.254-0.324) | 0.806 | -0.027(-0.212-0.158) | 0.769 | -0.084(-0.315-0.147) | 0.465 | 0.107(-0.117-0.331) | 0.337 |
| LDL-C(mmol/L) | 58 | 0.012(-0.111-0.135) | 0.843 | -0.043(-0.121-0.036) | 0.275 | -0.002(-0.100-0.096) | 0.966 | 0.000(-0.095-0.095) | 0.998 |
| HDL-C(mmol/L) | 58 | -0.127(-0.351-0.096) | 0.254 | 0.039(-0.104-0.182) | 0.578 | -0.067(-0.246-0.111) | 0.447 | 0.001(-0.172-0.174) | 0.989 |

Abbreviations: IYCF, Infant and Young Child Feeding index; DDS, Dietary Diversity Score; h-PDI, healthful Plant-based Diet Index; EAT-Lancet, EAT-Lancet diet score; BMI, body mass index; WHtR, waist-to-height ratio; SBP, systolic blood pressure; DBP, diastolic blood pressure; TC, total cholesterol; TG, triglycerides; LDL-C, low-density lipoprotein cholesterol; HDL-C, high-density lipoprotein cholesterol.

All dietary measures were converted to z-scores within the trajectory sample prior to analysis. β represents the change in the outcome per 1-SD increase in dietary quality. Infant diet was assessed using the IYCF index (2004–2008); school-age diet was assessed using DDS (2012–2013); adolescent diet was assessed using DDS (2016); adult diet was assessed using DDS, h-PDI, or EAT-Lancet score (2024). All four life-stage dietary exposures were entered simultaneously in a single OLS regression model, with the same covariates as the main analysis: treatment group, maternal and paternal education and occupation, household wealth index, sex, birth weight, gestational age, infant feeding pattern, age at follow-up, and total energy intake. Binary outcomes were not included in the life-stage-specific analysis because the small number of events (e.g., MetS, n = 29) combined with the simultaneous inclusion of four life-stage exposures would result in unstable Firth estimates with very wide confidence intervals, limiting interpretability. The primary analysis already reports binary outcomes in the main manuscript (Tables 3 and 4).

**Table S11.** Post-hoc Power Analysis for the Association Between Dietary Quality Trajectory and Metabolic Outcomes (*N* = 298).

| Outcome | Adjusted OR (95% CI) | *P*-value | n (Increasing) | n (Declining) | Prevalence (Declining) | Implied Prevalence (Increasing) | Post-hoc Power | *N* Required for 80% Power |
| --- | --- | --- | --- | --- | --- | --- | --- | --- |
| Overweight/obesity | 0.34 (0.15–0.77) | 0.009 | 131 | 102 | 0.275 | 0.114 | 0.88 | 184 |
| High WHtR | 0.44 (0.18–1.06) | 0.067 | 131 | 102 | 0.225 | 0.113 | 0.63 | 346 |
| MetS | 0.35 (0.07–1.70) | 0.195 | 131 | 102 | 0.059 | 0.021 | 0.32 | 854 |
| Metabolic problems | 1.24 (0.67–2.30) | 0.490 | 158 | 140 | 0.200 | 0.237 | 0.12 | 3986 |
| Elevated BP/hypertension | 0.99 (0.53–1.85) | 0.972 | 129 | 101 | 0.525 | 0.522 | 0.05 | —^a^ |

Abbreviations: OR, odds ratio; CI, confidence interval; MetS, metabolic syndrome; BP, blood pressure; WHtR, waist-to-height ratio.

Post-hoc power was calculated using two-proportion z-tests (two-sided α = 0.05). The prevalence in the declining (reference) trajectory group was obtained from the observed data. The implied prevalence in the increasing group was derived from the adjusted OR: p₁ = OR × p₂ / (1 − p₂ + OR × p₂). Power estimates are approximate, as the two-proportion test does not account for covariate adjustment; however, the adjusted OR reflects the effect of primary interest.Adjusted ORs and 95% CIs are from Table 4. All models were adjusted for parental occupation, parental education, household wealth, treatment group, birth weight, moderate physical activity, sleep quality, gender, smoking history, employment status, and age. MetS was analyzed using Firth penalized logistic regression; all other outcomes were analyzed using standard logistic regression.N required for 80% power was calculated as: *N* = 2 × [(z₀.₀₅ + z₀.₂₀)² × (p₁(1−p₁) + p₂(1−p₂))] / (p₁ − p₂)².

^a^: Not applicable (adjusted OR ≈ 1.0, indicating no meaningful effect to detect).

**Table S12**. STROBE Statement—Checklist of items that should be included in reports of cohort studies

|  | Item No | Recommendation | Page No |
| --- | --- | --- | --- |
| Title and abstract | 1 | (*a*) Indicate the study’s design with a commonly used term in the title or the abstract | 1 |
|  |  | (*b*) Provide in the abstract an informative and balanced summary of what was done and what was found | 1–2 |
|  | | | |
| Background/rationale | 2 | Explain the scientific background and rationale for the investigation being reported | 2–3 |
| Objectives | 3 | State specific objectives, including any prespecified hypotheses | 3 |
|  | | | |
| Study design | 4 | Present key elements of study design early in the paper | 3 |
| Setting | 5 | Describe the setting, locations, and relevant dates, including periods of recruitment, exposure, follow-up, and data collection | 3 |
| Participants | 6 | (*a*) Give the eligibility criteria, and the sources and methods of selection of participants. Describe methods of follow-up | 3; 18 |
|  |  | (*b*) For matched studies, give matching criteria and number of exposed and unexposed | N/A |
| Variables | 7 | Clearly define all outcomes, exposures, predictors, potential confounders, and effect modifiers. Give diagnostic criteria, if applicable | 3–5 |
| Data sources/ measurement | 8* | For each variable of interest, give sources of data and details of methods of assessment (measurement). Describe comparability of assessment methods if there is more than one group | 3–5 |
| Bias | 9 | Describe any efforts to address potential sources of bias | 6; 10–11 |
| Study size | 10 | Explain how the study size was arrived at | 3 |
| Quantitative variables | 11 | Explain how quantitative variables were handled in the analyses. If applicable, describe which groupings were chosen and why | 5–6 |
| Statistical methods | 12 | (*a*) Describe all statistical methods, including those used to control for confounding | 5–6 |
|  |  | (*b*) Describe any methods used to examine subgroups and interactions | 6 |
|  |  | (*c*) Explain how missing data were addressed | 6 |
|  |  | (*d*) If applicable, explain how loss to follow-up was addressed | 6; 18 |
|  |  | (*e*) Describe any sensitivity analyses | 6 |
| Results | | |  |
| Participants | 13* | (a) Report numbers of individuals at each stage of study—eg numbers potentially eligible, examined for eligibility, confirmed eligible, included in the study, completing follow-up, and analysed | 7; 18 |
|  |  | (b) Give reasons for non-participation at each stage | 3; 18 |
|  |  | (c) Consider use of a flow diagram | 18 |
| Descriptive data | 14* | (a) Give characteristics of study participants (eg demographic, clinical, social) and information on exposures and potential confounders | 7; 19–23 |
|  |  | (b) Indicate number of participants with missing data for each variable of interest | 19–23 |
|  |  | (c) Summarise follow-up time (eg, average and total amount) | 3 |
| Outcome data | 15* | Report numbers of outcome events or summary measures over time | 7; 24–27 |
| Main results | 16 | (*a*) Give unadjusted estimates and, if applicable, confounder-adjusted estimates and their precision (eg, 95% confidence interval). Make clear which confounders were adjusted for and why they were included | 27–31 |
| Other analyses | 17 | Report other analyses done—eg analyses of subgroups and interactions, and sensitivity analyses | 6; 9 |
| Discussion | | | |
| Key results | 18 | Summarise key results with reference to study objectives | 8 |
| Limitations | 19 | Discuss limitations of the study, taking into account sources of potential bias or imprecision. Discuss both direction and magnitude of any potential bias | 10–11 |
| Interpretation | 20 | Give a cautious overall interpretation of results considering objectives, limitations, multiplicity of analyses, results from similar studies, and other relevant evidence | 8–11 |
| Generalisability | 21 | Discuss the generalisability (external validity) of the study results | 10–11 |
| Other information | | | |
| Funding | 22 | Give the source of funding and the role of the funders for the present study and, if applicable, for the original study on which the present article is based | 32 |

*Give information separately for exposed and unexposed groups.

**Note:** An Explanation and Elaboration article discusses each checklist item and gives methodological background and published examples of transparent reporting. The STROBE checklist is best used in conjunction with this article (freely available on the Web sites of PLoS Medicine at http://www.plosmedicine.org/, Annals of Internal Medicine at http://www.annals.org/, and Epidemiology at http://www.epidem.com/). Information on the STROBE Initiative is available at http://www.strobe-statement.org.
